# Optoacoustically augmented magnetic guidewire for radiation-free minimally invasive therapies

**DOI:** 10.1126/sciadv.aea0201

**Published:** 2026-02-04

**Authors:** Fan Wang, Xianqiang Bao, Erdost Yildiz, Yan Yu, Xosé Luís Deán-Ben, Wenbin Kang, Shuaizhong Zhang, Devin Sheehan, Ren Hao Soon, Jelena Zinnanti, Daniel Razansky, Metin Sitti

**Affiliations:** ^1^ Physical Intelligence Department, Max Planck Institute for Intelligent Systems, 70569 Stuttgart, Germany.; ^2^ Institute for Biomedical Engineering, Department of Information Technology and Electrical Engineering, ETH Zurich, 8093 Zürich, Switzerland.; ^3^ Jiangsu Provincial Joint International Research Laboratory of Medical Information Processing, School of Computer Science and Engineering, Southeast University, Nanjing 210096, China; ^4^ Key Laboratory of New Generation Artificial Intelligence Technology and Its Interdisciplinary Applications (Southeast University), Ministry of Education, Nanjing 210000, China.; ^5^ Institute of Pharmacology and Toxicology and Institute for Biomedical Engineering, Faculty of Medicine, University of Zurich, 8057 Zurich, Switzerland.; ^6^ Department of Mechanical Engineering, City University of Hong Kong, 999077 Hong Kong, China.; ^7^ School of Mechanical Engineering, Yanshan University, Qinhuangdao, 066004, China.; ^8^ School of Medicine and College of Engineering, Koç University, 34450 Istanbul, Turkey.

## Abstract

Endovascular interventions are essential for treating cerebrovascular diseases, yet their monitoring methods commonly rely on ionizing radiation and contrast agents, posing unnecessary risks to patients and clinicians. We present a multifunctional optoacoustically augmented magnetic guidewire (OptoMaG) that integrates optoacoustic imaging with magnetic navigation to enable radiation-free, image-guided interventions. The ~250-micrometer flexible guidewire incorporates a 460-nanometer luminescent core with an enhanced optoacoustic signature and a FePt magnetic tip for precise, steerable control. Proof-of-concept studies show that OptoMaG can be actively navigated with external magnetic fields to traverse a 3D human-scale cerebrovascular phantom and accurately reach target brain sites. Beyond navigation, the FePt tip enables localized thermal ablation under remote radiofrequency stimulation, highlighting its theranostic potential for tumor treatment. In addition, OptoMaG functions as a light source for photodynamic therapy, selectively activating photosensitizers to destroy tumor cells while preserving healthy tissue. Collectively, OptoMaG provides a safe, radiation-free platform merging real-time navigation with targeted therapeutic capabilities.

## Teaser

OptoMaG combines magnetic navigation and optoacoustic imaging to enable radiation-free intravascular guidewire interventions.

## INTRODUCTION

Endovascular radiological interventions have become a mainstream method for treating cardiovascular diseases (*1*, *2*). During the procedure, the surgeon often uses one or more medical imaging modalities, such as fluoroscopy, computed tomography (CT), ultrasound (US) imaging, and magnetic resonance imaging (MRI), to guide a catheter or guidewire to the target sites (table S1) (*3*). X-ray fluoroscopy is now the gold standard imaging technique for endovascular interventions. These minimally invasive interventional radiological approaches not only reduce patient recovery time but also minimize the risks associated with traditional surgical procedures (*4*). Among the critical applications of interventional radiological therapy is the management of cerebrovascular diseases, which include conditions such as aneurysms, arteriovenous malformations, and ischemic strokes (*5*). The delicate and intricate nature of the brain vasculature poses substantial challenges, necessitating highly accurate and safe navigation of interventional instruments (*6*, *7*).

Guidewires and catheters are indispensable components of interventional procedures, functioning in a complementary manner. The guidewire typically serves as the initial navigational tool, enabling safe and precise access through complex and tortuous vascular pathways. Once the guidewire has reached the target location, the catheter is advanced over it to deliver therapeutic devices or agents (*8*). This coordinated use of guidewires and catheters is widely applied in various complex surgical interventions, including thrombectomy, coronary artery bypass grafting, and brain tumor treatments (*9*, *10*). The precise positioning and maneuvering are critical for the success of these procedures (*11*).

Interventional guidance predominantly relies on x-ray–based imaging, which, despite its efficacy, exposes both patients and medical personnel to ionizing radiation, raising concerns about its long-term health impacts (*12*). In addition, these methods often require the injection of contrast agents, which can cause patients to endure discomfort and potential side effects (*13*). In recent years, there has been a growing interest in developing ionizing radiation–free imaging techniques to enhance the safety profile of interventional procedures, improve surgical success rates, and provide patients with psychological reassurance (*14*). Optoacoustic (OA) imaging is an emerging hybrid imaging modality that uses non-ionizing short-pulsed laser energy in the near-infrared spectrum, thereby converting absorbed optical energy into US signals (*15*). This technique synergistically combines the rich optical contrast with the high spatial resolution of US in deep tissues, unaffected by photon scattering (*16*, *17*). It is known that the spectral dependence of optical absorption is closely associated with physiological properties, such as hemoglobin concentration and oxygen saturation. Consequently, OA imaging can generate detailed, high-contrast images of vascular structures without requiring ionizing radiation or contrast agents (*18*).

In this study, we report an optoacoustically augmented magnetic guidewire (OptoMaG) system to explore the integration of OA imaging technology into the navigation of guidewires for non-ionizing radiation–targeted therapy. The flexible OptoMaG with a diameter of 250 μm consists of a blue-light fiber and a magnetic guidance head and is coated with a polyvinylpyrrolidone (PVP) layer to enhance hydrophilicity and reduce friction against the vascular walls. Commercially available guidewires, such as nitinol or stainless steel coated with polymer layers, are designed for traditional imaging technologies and do not exhibit sufficient OA imaging contrast. Our multifunctional composite guidewire has been uniquely designed using ZnS:Cu particles to enhance such contrast. Meanwhile, the FePt-based magnetic head allows for precise directional control via magnetic fields and also generates heating via wireless application of radiofrequency (RF) waves to ablate targeted tumor sites. In addition, OptoMaG can emit and guide blue light, which can be precisely delivered to brain tumors to serve as a light source for photodynamic therapy (PDT). The OptoMaG system thus establishes a multifunctional therapeutic platform that integrates real-time navigation, thermal ablation, and PDT. As a non-ionizing alternative to conventional imaging modalities, it holds great promise in reducing health risks and psychological stress associated with ionizing radiation, thereby enhancing the safety and clinical applicability of minimally invasive procedures.

## RESULTS

### Conceptual design and fabrication of OptoMaG

**Fig. 1.**
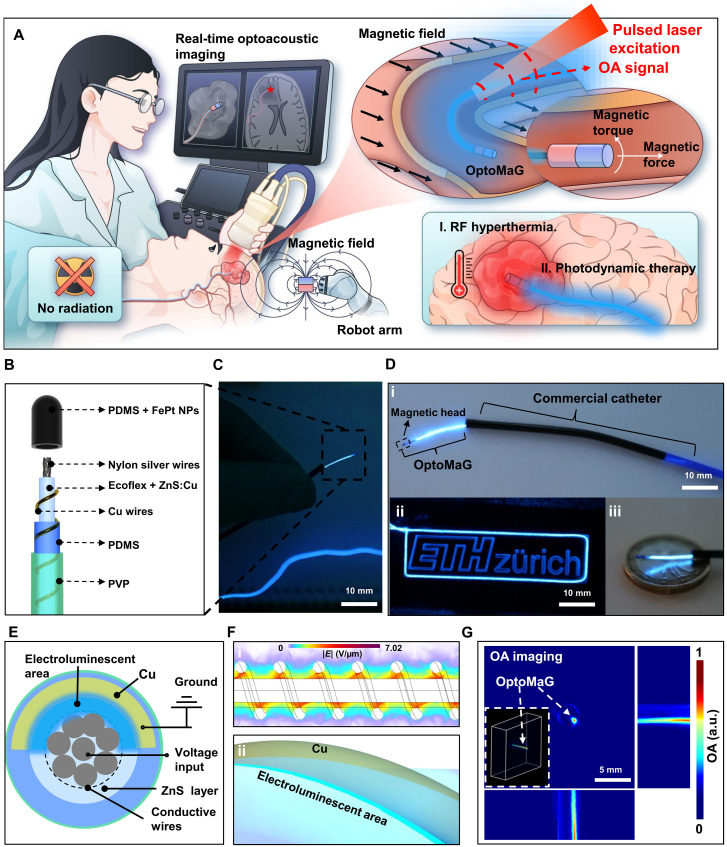
Radiation-free navigation of OptoMaG under 3D OA imaging. (**A**) Schematic of the guidewires guided by OA imaging, steered by magnetic fields, and eliminating deeply seated tumors with RF heating and PDT. Designed in Adobe Illustrator. (**B**) Schematic illustration of the OptoMaG comprising a magnetic head, a conductive core, a luminescent layer, a twisted conductive wire, a protective layer, and a thin biocompatible hydrogel outer layer. (**C**) The guidewire is held by two fingers. (**D**) Photograph of (i) the guidewire with a commercial catheter, (ii) the guidewires lighting the ETH Zurich logo, and (iii) size comparison with a 1€ coin (diameter = 23.25 mm, thickness = 2.33 mm). (**E**) Cross-sectional schematic diagram of the luminescent guidewire. (**F**) COMSOL simulation of electric potential distribution and schematic diagram of the luminescent region of the ZnS layer and the copper wire electrode in the luminescent guidewire. (**G**) 3D OA image of OptoMaG. a.u., arbitrary units.

To eliminate the need for ionizing radiation in interventional procedures under imaging modalities such as x-ray fluoroscopy for real-time monitoring, we developed OptoMaG to enable real-time 3D navigation and monitoring without ionizing radiation exposure (Fig. 1A). OptoMaG consists of a hard-magnetic FePt-based tip and an ~250-μm-diameter blue-emitting electroluminescent fiber (Fig. 1B). The magnetic tip is formed by embedding FePt nanoparticles in polydimethylsiloxane (PDMS). Since the FePt nanoparticles are hard magnetic materials with high coercivity and stable magnetization, they enable OptoMaG to be actuated via an external magnetic field for precise steering while also responding to remote RF heating for ablation procedures (*19*). The luminescent fiber segment features a central conductive nylon-silver fiber serving as the positive electrode, ZnS:Cu phosphor (see text S1 in the Supplementary Materials) mixed in Ecoflex polymer as the electroluminescent layer, a surface-wrapped copper wire as the grounding electrode, and a PDMS coating for waterproof and a surface layer of PVP are applied to enhance hydrophilicity and reduce friction. OptoMaG is designed to navigate with the help of commercially available catheters, and it can also be steered by a magnetic field to guide the catheters (Fig. 1, C and D i). After turning on the electroluminescent OptoMaG (Fig. 1D, ii and iii), the surrounding bright light from the OptoMaG guidewire illuminates the ETH Zurich logo and a 1-euro coin. An alternating voltage (Vpp = 10 V) applied between the Ag-nylon and copper electrodes drives a microampere-scale current to power the units (Fig. 1E). Electric field–induced excitation of the luminescent center and recombination of electron-hole pairs result in light emission from the ZnS:Cu phosphor at the weft-warp contact area (Fig. 1F). By varying the input voltage and frequency of the power source, we could accurately tune the luminance of the electroluminescent unit. Meanwhile, OptoMaG exhibits strong OA contrast due to the highly efficient light absorption and excellent photothermal conversion properties of the ZnS:Cu particles (Fig. 1G), thereby allowing physicians to accurately visualize the guidewire position and shape within the human body.

**Fig. 2.**
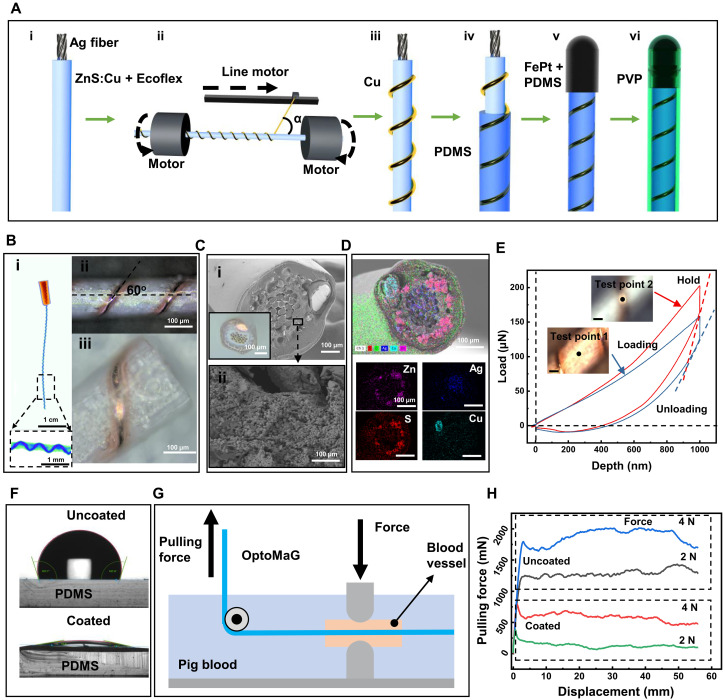
Fabrication process and characterization of OptoMaG. (**A**) Schematic illustration of the OptoMaG fabrication process: (i) coating luminescent material on Ag Nylon fibers, (ii and iii) winding copper wires, (iv) applying a PDMS layer for waterproofing, (v) attaching the magnetic tip, and (vi) coating with PVP polymer. (**B**) Micro-CT (i) and optical images (ii and iii) of OptoMaG from a top-down view. (**C**) SEM and optical images showing the cross section of OptoMaG (i), and ZnS:Cu particles distributed in the Ecoflex polymer (ii). (**D**) Elemental distribution (Zn, Ag, S, and Cu) across the OptoMaG cross section. (**E**) The nanoindentation curves of the OptoMaG on the surface of various locations. (**F**) Comparison of contact angles between coating PVP and uncoating PVP on the surface of PDMS. (**G**) Schematic diagram illustrating the friction measurement between OptoMaG and a blood vessel. (**H**) Comparison of the friction between coating PVP and uncoating PVP.

In our study, we engineer OptoMaG by first coating commercial Ag-nylon fibers with a ZnS:Cu mixture embedded in an Ecoflex polymer to form a luminescent layer (Fig. 2A i and fig. S1A i), followed by precise filament winding of copper wires around the fibers (Fig. 2A, ii and iii). Subsequent encapsulation with PDMS (Fig. 2Aiv and fig. S1Aii) and the integration of FePt nanoparticles imparted magnetic properties to the guidewires, facilitating their manipulation under an external magnetic field (Fig. 2Av and fig. S1B) and coating with a PVP layer to enhance hydrophilicity and reduce friction against the vascular walls (Fig. 2Avi). Using this process, we successfully fabricated the OptoMaG up to 10 m in length (fig. S1C).

### Material characterization of OptoMaG

After the fabrication process, we verified that the OptoMaG structure aligned with the design depicted in Fig. 2A. Micro-CT and laser microscopy revealed the structural details, including copper wires wound at a 60° angle and a white part representing the luminescent layer (Fig. 2B, i to iii). The cross-sectional structure of OptoMaG was analyzed using laser microscopy and scanning electron microscopy (SEM), as shown in Fig. 2 (C and D), clearly identifying the copper wires, Ag-nylon fibers, and the ZnS:Cu luminescent layer coating. Energy-dispersive spectroscopy mapping images (Fig. 2D and fig. S1, E and F) revealed the distribution of Zn and S within the luminescent layer. Ag was predominantly concentrated at the center of the cross section, corresponding to the conductive Ag-nylon fibers, while Cu was located in the copper wires and partially distributed in the luminescent layer.

The OptoMaG must have appropriate mechanical properties, particularly in terms of elastic modulus and friction between the guidewire and the vessel wall, to enable flexible navigation through blood vessels while minimizing the risk of vascular perforation. Figure 2E shows representative load-displacement curves obtained by performing five repeated indentations at two distinct locations on the OptoMaG using a Berkovich tip. The nanoindentation results revealed that the elastic modulus of the OptoMaG was 95.46 ± 17.2 MPa at point 1 and 125.26 ± 13.4 MPa at point 2. The corresponding hardness values were 8.35 ± 2.6 MPa and 11.2 ± 1.6 MPa at point 1 and point 2, respectively. In addition, the substantially lower elastic modulus of OptoMaG compared to silica fibers is anticipated to improve mechanical compatibility with soft tissue (*20*). In practical clinical applications, the relatively low elastic modulus of OptoMaG can be compensated for by using commercially available catheters with appropriate support characteristics. This approach is demonstrated in our subsequent experiments using a three-dimensional (3D) cerebrovascular phantom network.

PVP coating was applied to the OptoMaG surface, notably enhancing hydrophilicity and reducing friction to reduce friction between OptoMaG and the blood vessel. As shown in Fig. 2F, the contact angle changes from 137.4° to 12.5° after PVP coating, which shows that PVP coating can enhance the hydrophilicity of the PDMS surface. In addition, PVP coating can substantially reduce the friction between OptoMaG and the vessel wall. We also measured the forces required to pull OptoMaG with and without PVP coating at a constant speed (10 mm min^−1^) under different normal forces (2 and 4 N) applied by a pair of grips (Fig. 2G) (*21*). Our measurements showed that the self-lubricating PVP coating substantially reduced the pulling force (Fig. 2H). Under applied normal forces of 2 and 5 N, the PVP coating reduced the pulling force by 7.0-fold (from 1048.8 to 149.6 mN) and 3.1-fold (from 1798.1 to 588.0 mN), respectively, compared to the uncoated condition. These findings demonstrate the effectiveness of the self-lubricating PVP coating in reducing surface friction under load.

### Optical characterization of OptoMaG

**Fig. 3.**
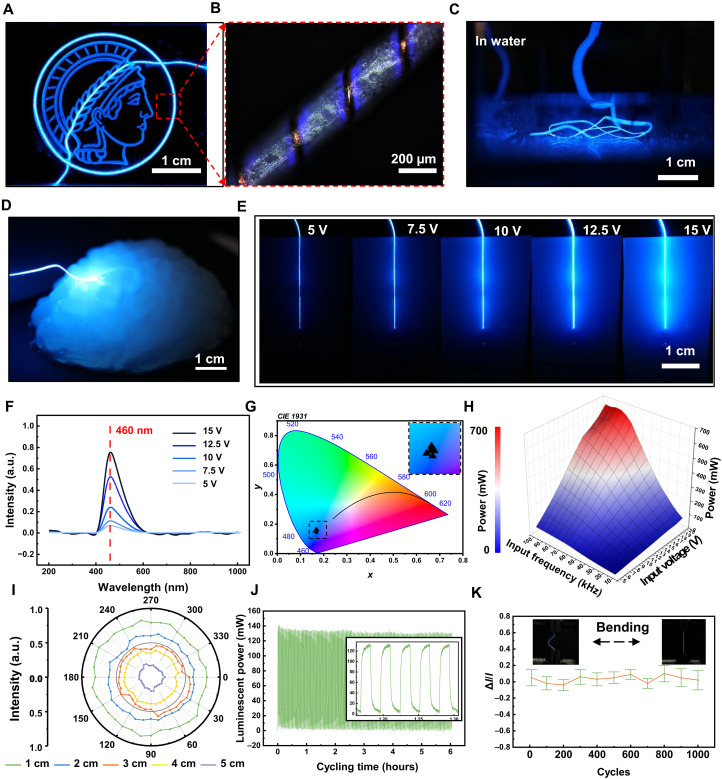
Luminescence performance characterization of OptoMaG. (**A**) Picture of a blue-luminescent guidewire implanted in an agarose brain model transmitting blue light to the deeper areas of the brain. Scale bar, 1 cm. (**B**) Picture of a head of the Minerva pattern lighted by a blue-luminescent guidewire. Scale bar, 1 cm. (**C**) Details of light around the copper electrode wires. Scale bar, 200 μm. (**D**)Picture of a blue-luminescent guidewire working under the water. Scale bar, 1 cm. (**E**) Picture of the different transmitting effects of a single blue-luminescent guidewire under different input voltages. (**F**) Electroluminescent spectra of a blue-luminescent guidewire with different input voltages. (**G**) The luminescence color of a blue-light guidewire in *x*, *y* chromaticity. (**H**) Luminescence power of a blue-luminescent guidewire under different input voltages and different frequencies. (**I**) Luminance distribution around the luminescent warp circumference. (**J**) Electroluminescence performance of a blue-light guidewire with 10,000 cycles of OFF-ON. (**K**) The luminance varied minimally when the blur-light guidewire was bent and released for 1000 cycles.

The optical performance of luminescent OptoMaG was first assessed independently ex vivo to verify its potential to provide a light source for PDT (*22*). A signal generator and an amplifier were used to provide the high-frequency power source to light the OptoMaG (fig. S2, A and B). Considering the bulkiness of the power supply system not suitable for clinical application, we designed a small and portable circuit as a power source for the OproMaG (fig. S2C). The blue-luminescent OptoMaG was embedded in an acrylic board with a Minerva logo channel, and the blue light can be transmitted to all the channels to light the Minerva logo pattern (Fig. 3A). The detailed examination of the guidewire surface reveals that the blue luminescence is concentrated around the copper wire electrode (Fig. 3B), consistent with the COMSOL simulation results of electric potential and electric field intensity (fig. S3, A and B). This phenomenon occurs because of the sufficient voltage potential established between the copper wire electrode and the Ag-nylon fiber electrode. When the voltage reaches a critical level, it induces the ZnS:Cu phosphor material embedded in the guidewire to emit light (*23*). We submerged the OptoMaG in water for 24 hours (Fig. 3C), where it continued to emit light, indicating its operational stability in aqueous environments such as blood vessels or soft brain tissue in the human body. As shown in Fig. 3D, the OptoMaG can transmit blue light to an agarose artificial brain, using the portable circuit as a power source. The subsequent images demonstrate the intensity of luminescence as a function of applied voltage, ranging from 5 to 15 V, with a noticeable increase in brightness correlating with higher voltages (Fig. 3E).

The frequency and emission wavelength of the optical signal emitted by the luminescent guidewire are generally influenced by the excitation signal. However, as demonstrated in the analysis, increasing the input voltage from 5 to 15 V does not induce a blue shift in the dominant wavelength of the emission spectrum, which remains stable at ~460 nm (Fig. 3F). This stability in the emission wavelength, despite changes in input voltage, indicates that the luminescence mechanism is primarily governed by the intrinsic properties of the ZnS phosphor material, rather than the variations in excitation voltage (*24*). The 460-nm wavelength offers critical advantages in PDT, particularly in the treatment of malignant tumors using 5-aminolevulinic acid (5-ALA) (*25*). To characterize the luminescence of OptoMaG, we measured the chromaticity coordinates of its emitted blue light of OptoMaG on a CIE 1931 color space diagram (*26*), highlighting the precise color characteristics of the luminescence (Fig. 3G). The luminescence power of OptoMaG can be influenced by both the input voltage and the input frequency.

We also measured the luminescence power intensity distribution of 30-cm OptoMaG powered by different voltages and frequencies, as depicted in the 3D plot in Fig. 3H. At an input signal of 80 kHz and 14 V, the luminescence power reaches up to 690 mW, providing sufficient light intensity for PDT applications (*27*). This control over luminescence power allows for precise adjustment of light intensity, which is critical in managing the penetration depth of light within human tissues. The luminance distribution around the circumference of the luminescent guidewire, as shown in Fig. 3I, demonstrates that OptoMaG can transmit wireless optical signals omnidirectionally, with nearly identical signal intensity detected in all directions. This uniform illumination is critical for ensuring consistent visibility and therapeutic effectiveness, regardless of the OptoMaG’s orientation.

The signal generator was also programmed to change the period and on-off mode (fig. S3, C and D, and movie S1). When the input voltage is 10 V, the short current was measured at different frequencies from 10 to 100 kHz (fig. S3, E to G). This correlation between the voltage potential and light emission underscores the efficient design of the luminescent guidewire, where precise electrical control can modulate the luminescent output (*28*). As shown in fig. S3H, the temperature change measured by the infrared thermal camera was ~0.4°C. By fine-tuning the input voltage and frequency, the emitted light can be delivered with sufficient power to effectively treat targeted tumor tissues while minimizing the risk of damaging surrounding healthy tissue. This tunability of light intensity ensures that the treatment is both effective and safe, optimizing therapeutic outcomes in PDT applications.

Furthermore, Fig. 3J shows that after 10,000 cycles of ON-OFF switching (fig. S3, D and H), the blue-light-emitting guidewire maintains stable luminance intensity with less temperature change, underscoring its robust performance and long-term reliability. Such stability is essential for applications requiring continuous and repeatable operation. Moreover, Fig. 3K reveals that the guidewire exhibits minimal variation in luminance even after 1000 cycles of bending and release. This result indicates that the guidewire not only retains its mechanical flexibility but also maintains stable optical output, confirming its suitability for dynamic environments where both durability and consistent light emission are critical.

### Active steering and navigation through a 3D cerebrovascular phantom network

**Fig. 4.**
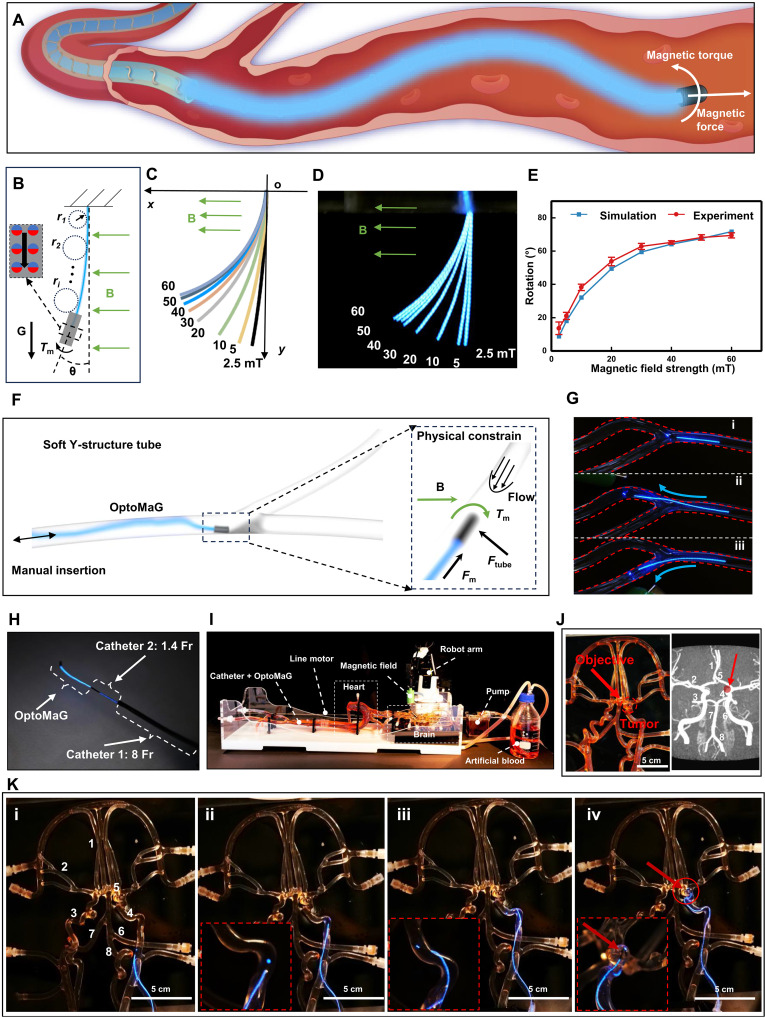
Active steering and navigation through a 3D cerebrovascular phantom. (**A**) A soft luminescent guidewire with a magnetically responsive tip with uniform magnetization (*M*) and a tapered FePt NPs core required for active steering is steered by magnetic torque (*T*_m_), induced from the external magnetic field (*B*). Designed in Adobe Illustrator. (**B**) Deformation of OptoMaG under varying magnetic field strengths. (**C**) Simulated shapes of OptoMaG at different magnetic field intensities. (**D**) Luminescent OptoMaG responds to different magnetic field strengths in real time. (**E**) Comparison of deformation angles between simulation and experimental results, showing OptoMaG’s shape at four different magnetic field strengths. (**F**) A Y-shaped soft tube is used to demonstrate OptoMaG’s ability to selectively navigate through the correct channel under magnetic field control while moving upstream against the flow. OptoMaG is manually inserted, and the schematic depicts the physical constraints within the tube. (**G**) Images showing the luminescent OptoMaG intelligently selecting different channels in response to varying magnetic fields. (**H**) Two different-sized catheters are used to guide the luminescent OptoMaG to the target area. An 8 Fr catheter navigates close to the brain vasculature, followed by a 1.4 Fr catheter, which directs OptoMaG to the targeted tumor location. (**I**) Active steering and navigation of OptoMaG through a 3D cerebrovascular phantom network filled with flowing artificial blood. (**J**) Comparison of the 3D cerebrovascular phantom network and the magnetic resonance angiography of the circle of Willis. 1, anterior cerebral artery; 2, middle cerebral artery; 3, internal carotid artery; 4, posterior communicating artery; 5, ACOM; 6, posterior cerebral artery; 7, basilar artery; 8, vertebral artery (Reproduced from Wikimedia Commons, https://w.wiki/HZQ9, CC-BY-3.0). (**K**) Sequential images of the OptoMaG navigating from the right carotid artery to the brain tumor under telerobotic magnetic steering.

Magnetic actuation systems were used for the remote control and steering of the OptoMaG, as shown in Fig. 4A. When a magnetized object, such as a microrobot, is exposed to an externally applied magnetic field, it can experience magnetic force *F*_m_ and torque *T*_m_ within the region of magnetic fields (see text S2 in the Supplementary Materials). Under the influence of an external magnetic field *B*, the magnetic tip experiences a magnetic torque, resulting in reversible bending deformation (Fig. 4B). This controllable bending can be used to guide the guidewire along the correct path, demonstrating its potential for precise manipulation. To achieve bending deformations under varying magnetic fields that satisfy practical clinical operational requirements, we conducted simulations. Finite element analysis was performed using ABAQUS, following the methodology outlined in the previous study (*21*). The magnetic moment of the OptoMaG head is 1.059 × 10^−5^ A·m^2^. We applied magnetic fields of 2.5, 5, 10, 20, 30, 40, 50, and 60 mT, respectively (movie S2). To simplify the model, OptoMaG was vertically suspended (Fig. 4B) with the gravitational force acting downward and the applied magnetic field oriented horizontally to the left. The resulting deformations under different magnetic fields were obtained and consolidated into a single figure (Fig. 4C). In addition, we conducted experiments to measure the deformation of OptoMaG under various magnetic fields. OptoMaG was vertically suspended within the working region of a vibrating sample magnetometer (EZ7, MicroSense, USA), which generated a uniform magnetic field corresponding to the simulations. The deformation induced by different magnetic field strengths was recorded and consolidated into a single figure (Fig. 4D). To quantify the deformation of OptoMaG, we selected two parameters: bending angle θ and the minimum radius of curvature *r*_min_ (Fig. 4B). The bending angle is defined as the angle between the OptoMaG tip and the vertical direction. The outer contour of OptoMaG was segmented into multiple sections, and the radius of curvature *r_i_* for each segment was determined using MATLAB fitting. The smallest *r_i_* was designated as the minimum radius of curvature for the contour. We conducted statistical analyses of the deformation obtained from both simulations and experiments, resulting in a comparative plot of the corresponding bending angles and minimum radii of curvature (Fig. 4E and fig. S4A). The results demonstrated excellent consistency between the simulated and experimentally measured deformation.

We conducted experiments using a Y-shaped soft vessel model to evaluate OptoMaG’s ability to autonomously select a path and navigate against fluid flow (Fig. 4F). OptoMaG was introduced into the Y-shaped conduit and manually advanced while an external magnetic field, generated by a robot arm–controlled permanent magnet, guided its movement. We simulated magnetic cubes of different sizes, and the 100 mm by 100 mm by 100 mm magnetic cube was able to achieve a penetration depth of 130 mm at a magnetic field exceeding 10 mT (fig. S4, B to F), providing sufficient depth to control the OptoMaG within the human body in clinical application (*7*). The navigation process was influenced by a combination of forces, including fluid impact, wall interactions, friction, and externally applied magnetic torques and gradients. By adjusting the external magnetic field, OptoMaG was directed into either the upper or lower channel of the Y-structure (Fig. 4G and movie S3). Unlike conventional magnetic pulling–based approaches, the steering mechanism leveraged both magnetic torque and gradient forces, enabling controlled directional changes rather than simple attraction.

We used the example of an intracranial aneurysm intervention surgery to further demonstrate OptoMaG’s precise navigation ability under the influence of an external magnetic field and verify its potential for PDT at the aneurysm site. Because of the tortuous and complex nature of cerebral blood vessels, we used a 1.6 French scale (Fr) commercial neuro catheter to assist in guiding OptoMaG to the target location under the control of an external magnetic field (Fig. 4H). The vascular model contained artificial blood, and a pump was used to drive the artificial blood to simulate blood flow conditions (Fig. 4I). The magnetically responsive tip of the OptoMaG, which is embedded with magnetic nanoparticles, enables precise steering via an actuating magnet at the end-effector of a robotic arm. The OptoMaG is also compatible with standard microcatheters, which can travel over the guidewire along the navigated path. The OptoMaG was inserted into a 1.4 Fr catheter, which was then placed inside a larger 8 Fr catheter (Fig. 4H). The assembled catheter was placed into the whole-body vascular model (SJX014) and advanced by a linear motor. As shown in Fig. 4J, we selected the anterior communicating artery (ACOM) as the targeted area, which is the most common site of intracranial aneurysms (*29*). Figure 4K and movie S4 illustrate the entire experimental procedure. In straight blood vessels, OptoMaG continuously advanced along the vessel (Fig. 4Ki). Upon encountering a vascular bend (Fig. 4Kii), the robotic arm adjusted the position of the magnet proximity, causing the tip of OptoMaG to bend under the influence of the magnetic field (Fig. 4Kiii), thereby achieving the correct direction of movement. Then, OptoMaG was continuously pushed forward, successfully navigating the curved section. It continued to advance and ultimately reached the target location (Fig. 4Kiv). Before reaching the cerebral vasculature, OptoMaG traverses through the cardiovascular system from the right femoral artery to the brain, aided by the rigidity of the 8 Fr catheter. The thinner 1.4 Fr catheter is essential for navigating the more tortuous brain vessels, minimizing the risk of vessel perforation. Once at the target location, OptoMaG can use its light source to emit light at a wavelength of 460 nm to activate photosensitive drugs, achieving PDT. This experiment not only validates OptoMaG’s controllable navigation ability in complex vascular environments but also demonstrates its potential for minimally invasive intervention treatments, providing technological support for future precise treatment of intracranial aneurysms.

### Real-time OA imaging of the OptoMaG in vitro and ex vivo

**Fig. 5.**
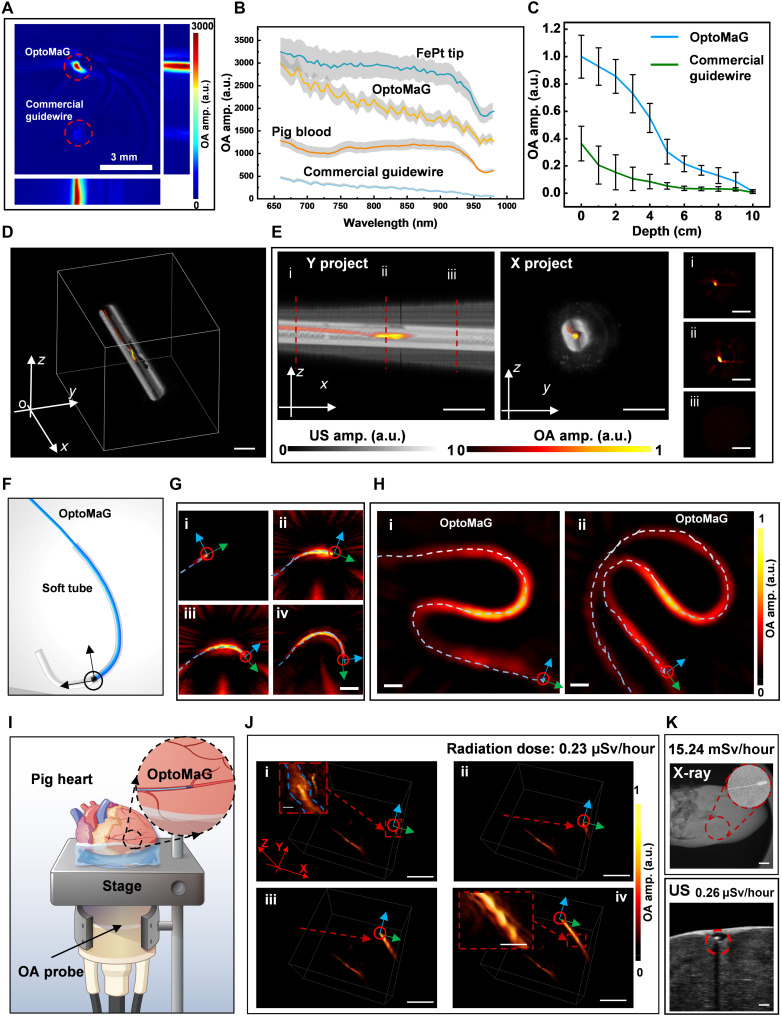
Characterization of OptoMaG navigation and monitoring with OA imaging in an ex vivo porcine heart model. (**A**) OA images of OptoMaG and commercial guidewire at 800-nm wavelength. (**B**) OA spectra of magnetic tips, OptoMaG, porcine blood, and commercial guidewire. (**C**) The normalized OA signal intensity generated by the OptoMaG and commercial guidewire at varying tissue depths. (**D** and **E**) Superimposition of OA and US images of OptoMaG with magnetic tips in a soft tube and the corresponding cross-sectional OA images (i and ii) are taken at the positions of the luminescent guidewire and magnetic tip, respectively, while (iii) denotes a location where OptoMaG is not present. Scale bars, 5 mm. (**F**) Schematic of guiding OptoMaG through the soft tube in deep tissues with external magnetic fields. (**G**) OA images of OptoMaG illustrate the migration of an MC in the C-structure model vessel. Scale bar, 5 mm. The thickness of the tissue above the OptoMaG is 10 mm. (**H**) OA navigation of OptoMaG through different patterned soft vessel models. Scale bars, 1 mm. The thickness of the tissue above the OptoMaG is 10 mm. (**I**) Schematic of the experimental setup for real-time OA imaging of OptoMaG moving through the vasculature of the porcine heart. Designed in Adobe Illustrator. (**J**) Ex vivo experiment of OptoMaG moving inside the porcine heart vessel and monitored by 3D real-time OA imaging. The magnified view in (i) shows the raw imaging data. Scale bars, 5 and 1 mm [magnified insets of (i) and (iv)]. (**K**) Comparative images of the OptoMaG taken with x-ray CT and US. Scale bar, 5 mm.

Compared to commercial guidewires composed of metal-coated Polytetrafluorethylen (PTFE) polymers, OptoMaG manifested substantially higher OA contrast (Fig. 5A and table S2), which persisted over a broad spectral range between 650 and 980 nm, also relative to the background contrast from whole blood (Fig. 5B). This enhancement is attributed to the higher absorbance of FePt nanoparticles compared to ZnS:Cu phosphor at the same concentration of 1.0 mg/ml (fig. S5A). Its superb OA contrast suggests that OptoMaG can be effectively imaged in deep tissues (see Fig. 5C), indicating its potential for real-time navigation and monitoring of clinical vascular procedures. The emitted light from OptoMaG could effectively penetrate through 3 to 4 mm of blood (fig. S5B) under an applied voltage of 10 V and a frequency of 50 kHz, which also demonstrates that OptoMaG can enable PDT in deep tissues.

We conducted both OA and US imaging experiments to assess the trackability of the fully assembled OptoMaG. For this, we used a volumetric OA tomography system to acquire 3D images of OptoMaG embedded in solidified agarose (Fig. 5D and fig. S5, F and G). As shown in Fig. 5E, OA signals were selectively detected from the luminescent components, while the surrounding agarose generated negligible signals, ensuring high imaging contrast. The *X*-axis projection further highlights the spatial distribution of the OA signals, reinforcing the precise localization capability of this technique. The right figures present cross-sectional images at positions “i,” “ii,” and “iii,” revealing variations in signal intensity corresponding to different structural features within the OptoMaG. The OptoMaG, shaped into a helical structure (fig. S5C), was accurately visualized in 3D through OA projections along the *ZY*, *ZX*, and *XY* planes (fig. S5, D and E), confirming its well-preserved spatial configuration.

To assess the feasibility of real-time tracking and precise navigation of OptoMaG in deep tissue-mimicking environments, we designed three different tissue-mimicking phantoms with tubings embedded under ~10 mm of agarose gel (Fig. 5, F to H, and fig. S5, C to G). The phantoms contained one, two, and three C-shaped bends to evaluate OptoMaG’s maneuverability. Under OA imaging guidance, OptoMaG successfully navigated through all the tubings with high tracking accuracy (movie S5), demonstrating its potential for minimally invasive interventions. Notably, OA signals remained detectable at a depth of 10 mm, ensuring reliable visualization.

Subsequently, we constructed an ex vivo porcine cardiovascular phantom and placed it on top of the spherical array probe to facilitate real-time OA visualization to evaluate the 3D real-time OA imaging performance of OptoMaG in biological tissues (Fig. 5I). Branched microvessels extracted from a porcine heart were perfused with fresh porcine blood using a continuous flow system to simulate physiological conditions. When OptoMaG was successfully inserted into the tubing connected to the vascular phantom and moved into the imaged field of view, its trajectory could be tracked and visualized in real-time in a 3D workspace (Fig. 5J and fig. S6A). Background subtraction in the multispectral optoacoustic tomography (MSOT) imaging process revealed the OptoMaG signal with enhanced contrast, enabling clear delineation of vascular structures against the blood background (fig. S6A). Furthermore, by applying a robot arm–controlled neodymium (NdFeB) permanent bar magnet, we demonstrated the precise and steerable magnetic control of OptoMaG within a Y-shaped vascular structure (fig. S6, B to D).

Furthermore, we systematically compared the imaging performance of OptoMaG with MRI, x-ray CT, and US (Fig. 5K and fig. S7), with a particular focus on radiation exposure and image fidelity. MRI struggled to visualize the magnetic tips of OptoMaG due to signal distortion and heating effects caused by the interaction of magnetic materials with high-frequency fields. Although x-ray CT operated at a radiation level of 15.24 Millisievert (mSv)/hour, it failed to provide clear vascular visualization without the use of contrast agents. While US imaging effectively captured OptoMaG, OA imaging offered superior contrast and resolution. When applied to in vivo biomedical applications, OA can also provide functional information on oxygenation, hemoglobin levels, and tumor-specific features.

To further assess the imaging performance of OptoMaG in deep tissues, we conducted ex vivo experiments using a freshly euthanized mouse. Since the size of OptoMaG was incompatible with the murine vascular system, we introduced it via the oral cavity and esophagus (fig. S8, A and B). OA imaging was then performed in the thoracic and abdominal regions, successfully detecting OptoMaG at three distinct locations (fig. S8C). In addition, real-time 3D imaging provided precise spatial localization of OptoMaG within the body (fig. S8D). These findings underscore OA’s capability to achieve high-resolution, real-time visualization of catheter-based interventions within biological tissues, paving the way for potential clinical applications.

### Minimally invasive therapy applications: RF hyperthermic and photodynamic therapies

**Fig. 6.**
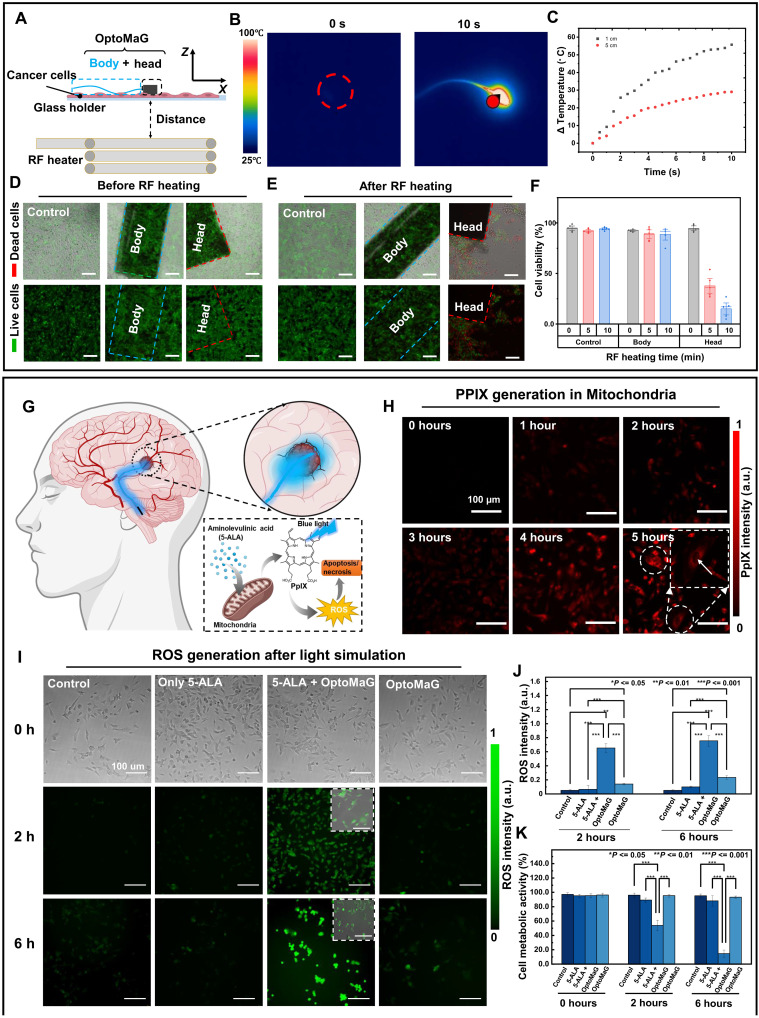
Minimally invasive therapy applications: RF hyperthermia and PDT. (**A**) Experimental setup for remote RF hyperthermia to kill the cancer cells. (**B**) Infrared temperature images of OptoMaG. (**C**) The temperature elevation in the magnetic tip of OptoMaG was induced by RF heating at distances of 1 and 5 cm. (**D**) Comparison of the conditions of three experimental groups of SH-SY5Y cells after 72 hours of culturing with RF heating. Scale bars, 100 μm. (**E**) The condition of three experimental groups of SH-SY5Y cells after various durations of RF heating. (**F**) SH-SY5Y cell survival in three groups after RF induction heating. (**G**) Schematic of PDT for the treatment of glioblastoma using OptoMaG, providing a blue light source. 5-ALA–PDT induces cell death via apoptosis/necrosis. 5-ALA accumulates in the mitochondria and forms protoporphyrin IX (PPIX) using the heme synthesis pathway. Activation with light of a specific wavelength causes a photodynamic reaction, producing ROS, which in turn leads to cell death. Designed in Adobe Illustrator. (**H**) 5-ALA–induced PPIX generation in the presence of 5-ALA or OptoMaG’s blue light. PPIX distribution in the cells can be visualized by its red fluorescence. (**I**) ROS generation after PDT on the SH-5SY distribution in the cells can be visualized by its red fluorescence. (**J**) Visualization of intracellular ROS 0, 2, and 6 hours (h) after OptoMaG’s blue light irradiation in SH-SY5Y. (**K**) SH-SY5Y cell survival after ALA-PDT in the presence of ALA or OptoMaG’s blue light.

To illustrate the noninvasive RF electromagnetic heating capabilities of OptoMaG, magnetic tips were positioned in proximity to tumor tissue (Fig. 6A). External high-frequency magnetic fields were used to heat these ferromagnetic materials, resulting in localized temperature increases that effectively kill cancer cells (*30*). We measured the hysteresis loop of FePt nanoparticles (fig. S9A), which exhibits a high remanence of up to 32.6 electromagnetic unit/g, ensuring that OptoMaG has good controllability under an external magnetic field. COMSOL simulations showed that during RF hyperthermia, OptoMaG’s magnetic tip can reach ~100°C within 30 s [see fig. S9 (B and C) and movie S6]. Experimental results confirmed that the magnetic tip heated to 82°C within 10 s, while the temperature increased by 55°C at a distance of 1 cm within 10 s (Fig. 6, B and C). This temperature is sufficient to kill tumor cells, while the heating time can be adjusted to regulate temperature rise and avoid damage to healthy tissue (*31*).

To test the OptoMaG’s ability to target and kill cancer cells via RF hyperthermia, in vitro experiments were conducted using the neuroblastoma cell line SH-SY5Y. First, we cultured cells in three groups: a control group, an OptoMaG group, and a magnetic tips group (fig. S9D). After 24 hours of incubation, all groups exhibited healthy cell growth (Fig. 6D). Notably, SH-SY5Y cells exposed to OptoMaG’s body and head maintained survival rates of 92.8 ± 1.3% and 94.8 ± 3.5%, respectively (Fig. 6, D and F), further indicating the good biocompatibility of OptoMaG. Next, RF heating was applied for 5 and 10 min, respectively. It was found that cells surrounding the magnetic tip were killed because of the RF-induced temperature rise of the magnetic tip (Fig. 6E). After 5 min of heating, only 37.8 ± 11.0% of the cells survived, and after 10 min, only 15.0 ± 8.7% of the cells remained viable (Fig. 6F).

In the second demonstration, OptoMaG was validated for PDT applications, which rely on the interaction of three components: a photosensitizer, light, and oxygen, to induce cell death through mechanisms of necrosis and apoptosis (*32*). While the visible light wavelengths typically used for PDT cannot penetrate deeply enough to reach deeper tumor locations, the flexible and controllable OptoMaG system can effectively navigate complex vascular systems to accurately reach deep-seated tumors in the brain. OptoMaG can emit blue light at a wavelength of 460 nm, which is ideal for activating the photosensitizer protoporphyrin IX (PPIX), which then produces superoxide and hydroxyl radicals, leading to cytotoxic effects. As the most widely used second-generation photosensitizing treatment, 5-ALA leads to specific overaccumulation of PPIX in tumor cells, leading to the generation of reactive oxygen species (ROS), which in turn leads to apoptosis and necrosis after OptoMaG irradiation (Fig. 6G).

To assess the effectiveness of OptoMaG’s 460-nm blue luminescence as a light source for 5-ALA–based PDT, we evaluated the intracellular accumulation of PPIX in SH-SY5Y neuroblastoma cells. As shown in Fig. 6H, the fluorescence intensity in the cells increased progressively over time, indicating continuous production and accumulation of PPIX. Fluorescence microscopy images taken 5 hours after 5-ALA treatment revealed that fluorescence was primarily localized in the cytoplasm, with minimal fluorescence observed in the nuclei (see the inset in Fig. 5H).

In PDT, cell death is primarily caused by oxidative damage resulting from increased production of ROS (*33*). To test this, we examined ROS generation and its cytotoxic effects under OptoMaG illumination, emitting at 460 nm with a light power of 100 mW. Cells treated with both 5-ALA and OptoMaG illumination exhibited visibly higher ROS fluorescence intensity compared to control groups, as visualized by fluorescence microscopy (Fig. 6I and fig. S10) and quantified using a microplate reader (Fig. 6J). Only the group treated with 5-ALA and exposed to OptoMaG blue light exhibited high fluorescence intensity, confirming that OptoMaG can induce ROS production in 5-ALA–based PDT, effectively killing cancer cells. ROS fluorescence intensity was further quantified using a microplate reader (485-nm excitation, 535-nm emission), consistent with the results observed under a fluorescence microscope (Fig. 6J). In addition, cell viability was assessed using the WST-8 cell counting kit. As shown in Fig. 6 (J and K), the group treated with both 5-ALA and OptoMaG blue light exhibited the highest ROS fluorescence intensity and the lowest cell viability. These findings further demonstrate that OptoMaG is effective for the 5-ALA–based PDT, providing foundational experimental evidence for future clinical research. These two application demos highlight OptoMaG’s potential for targeted, minimally invasive, and radiation-free cancer therapy through RF hyperthermia and PDT, enabling precise tumor destruction while preserving healthy tissues.

## DISCUSSION

The conventionally used x-ray–based tracking guidewires expose both patients and clinicians to harmful ionizing radiation, further necessitating contrast agents that cause patient discomfort. Conversely, the OptoMaG system integrates OA imaging into a flexible magnetic guidewire that involves no ionizing radiation. This approach not only enhances patient safety but also enables high-contrast, real-time visualization of vascular structures, facilitating improved navigation and positioning during interventions. OptoMaG’s magnetic head further contributes to its multifunctionality by offering precise directional control via external magnetic fields and enabling localized heating through RF waves, which can be used for therapeutic applications such as brain tumor ablation. In addition, the OptoMaG emits blue light at a 460-nm wavelength, which can be precisely delivered to the targeted brain tumors to serve as a light source for PDT.

X-ray fluoroscopy is now the gold standard imaging technique for endovascular interventions. To help interventionalists overcome various navigational challenges and reduce radiation exposure, efforts have been made to enhance guidewire functionality. These include mechanical models, artificial intelligence–assisted robotics, and magnetic materials for improved navigation (*34*). Although MRI represents a noninvasive and ionization-free medical imaging technique that uses powerful magnets to produce a strong magnetic field, conventional guidewires are metallic and not applicable in the MRI environment due to the risk of RF-induced heating, magnetic force, and severe image artifacts. US imaging, although useful for real-time navigation, often struggles with artifacts like shadowing, reverberations, and blooming caused by the strong reflections from metallic guidewires (*3*). Most research in this domain has focused on modeling and addressing basic mechanical or navigational challenges (*35*). However, there has been limited innovation in customizing guidewires for new emerging imaging techniques such as OA imaging; OA enables real-time 3D visualization with improved contrast, molecular imaging, and fewer artifacts, all without ionizing radiation (*36*). In our work, the guidewire is made of light-absorbing materials to enhance OA visibility and includes electrodes that activate ZnS:Cu phosphors, emitting 460-nm light for deep brain PDT, overcoming the limited penetration of conventional blue light and expanding therapeutic potential (*37*).

Although the OptoMaG system shows much promise, x-ray fluoroscopy remains the clinical gold standard for endovascular interventions. Hence, the OptoMaG system is not intended to replace existing guidewires but to inspire the development of next-generation guidewires with enhanced integration into emerging imaging pipelines. Despite promising initial proof-of-concept, the current results are largely based on in vitro experiments, necessitating comprehensive in vivo studies in animal models and clinical trials to assess the efficacy, safety, and potential risks of OptoMaG in diverse medical applications. The OptoMaG platform exhibits substantial potential for in vivo translation owing to its biocompatible design, wireless actuation, and non-ionizing imaging modality. The magnetic and optical stimuli used here fall within clinically accepted safety thresholds (safe magnetic field < 2 T, optical radiant exposure required for reliable stimulation is 0.34 to 0.48 J/cm^2^), suggesting feasibility for preclinical animal studies (*38**,* *39*). Future investigations will focus on validating its biostability, hemodynamic compatibility, and imaging performance in small-animal vascular and neurovascular models (*40*). Moreover, integrating near-infrared excitation could enhance penetration depth, while developing minimally invasive delivery catheters may enable precise deployment within in vivo neurovascular environments. These efforts will bridge the gap between in vitro proof of concept and preclinical feasibility, paving the way for translational applications of OptoMaG in image-guided neurointervention and targeted therapy.

One of the main obstacles to the clinical translation of OA imaging lies in its limited penetration depth, especially for transcranial applications. While OA imaging can be integrated with fluoroscopy to reduce radiation exposure and add depth-resolved vascular visualization during catheter navigation (*41*), achieving reliable imaging through the skull remains a major challenge due to acoustic attenuation and scattering (*42*). Recent advances in US beamforming and transducer array design offer potential solutions by focusing acoustic energy to improve signal detection across bone interfaces (*43**–**45*). Future efforts should prioritize integrating near-infrared–responsive materials into the OA platform to extend optical penetration, coupled with flexible array transducers (*46*) and optimized coupling layers to enhance acoustic transmission. Rather than relying solely on postprocessing algorithms (*47**,* *48*), engineering refinements in device design (*49*), such as optimizing catheter geometry and incorporating localized acoustic windows, represent more practical routes toward real-time transcranial OA guidance (*50*). These developments will be essential to unlock the full translational potential of OptoMaG for deep tissue and vascular interventions.

Furthermore, the particular OA imaging setup used in this study is limited by its relatively narrow field of view, hindering real-time visualization of the entire OptoMaG device (*51*). Addressing these technical limitations will require exploring novel nanomaterials and coatings to enhance durability, biocompatibility, and signal efficiency under various physiological conditions. We expect OA imaging technology to expand in the near future with more advanced optical guidance methods and US sensor arrays. Multidisciplinary collaboration will be key to refining OptoMaG for clinical translation, regulatory approval, and adoption. By providing a non-ionizing alternative, it could reduce the risks and anxiety associated with ionizing radiation, benefiting both patients and clinicians. Moving forward, overcoming current limitations and exploring new therapeutic possibilities will be pivotal in advancing OptoMaG for minimally invasive neurosurgery and cancer treatment, ultimately expanding its impact in medical science.

## MATERIALS AND METHODS

### Design and fabrication of OptoMaG

#### Fabrication of luminescent guidewire

Commercially available zinc sulfide phosphors (EL-8000, Shenzhen Yi Lai Technology Co.) were dispersed in Ecoflex 00-10 with a weight ratio of 3:1 by mixing with a planetary mixer (the revolution speed, rotation speed, and mixing duration are set to 640 rpm, 0 rpm, and 600 s, respectively) for 10 min. After degassing in a vacuum oven, the as-prepared mixtures were loaded on the silver-plated nylon yarns (100D, Hengtong X-silver Speciality Textile Co.) on a continuous producing line (Fig. 2Ai and fig. S1A). Silver-plated yarns were dipped into the zinc sulfide phosphor dispersions and passed through the center of a scraper ring with an inner diameter of 0.32 mm, followed by drying at 120°C in an air dry oven. The movement speed of the yarns was 0.1 m min^−1^. A coating process was conducted three times to prepare the luminescent fibers with a diameter of ~0.2 mm. Then, the 20-μm-diameter copper wires onto the ZnS-coated wire are shown schematically in Fig. 2Aii. The two ends of the ZnS-coated wire were fixed by two motors, and a spinnable copper wire was fixed onto a precisely motorized translation stage. Continuous, aligned copper wire was drawn out of the spinnable copper wire and attached to the modified ZnS-coated wire at an angle α. To achieve water resistance, the PDMS elastomer (Sylgard 184, Dow Inc.) was further dip-coated on the luminescent wires (Fig. 2A, ii to iv, and fig. S1A).

#### Fabrication of magnetic tips

FePt magnetic nanoparticles, synthesized as described in a previous study (*19*), were mixed with uncured PDMS elastomer to fabricate the magnetic tips for the guidewire. The mass ratio of magnetic nanoparticles to uncured silicone rubber (part A:B = 10:1) was maintained at 1:1. The mixture was homogenized using a planetary mixer to ensure uniform dispersion of the nanoparticles within the polymer matrix. The uncured magnetic soft composite was then poured onto a 3D-printed model, and a razor blade was used to flatten the surface. During this process, the blade was slid across the upper surface of the 3D model to remove excess material. This flattening step was repeated until a smooth, uniform surface was achieved. The laser-drilled acrylic was then aligned with the 3D model, and the guidewire was threaded through the preformed holes and immersed in the uncured magnetic soft composite. The assembly was cured in a 90°C oven for 2 hours. After curing, the mold and acrylic sheet were removed to obtain the guidewire with integrated magnetic tips (Fig. 2Av and fig. S1B).

#### PVP coating

To enhance the hydrophilicity of the guidewire and reduce friction with the vessel walls, the prepared guidewire was soaked in a PVP (10 mg/ml; molecular weight:, 360,000; Sigma-Aldrich) solution for 12 hours. After soaking, it was dried at room temperature to obtain the OptoMaG (Fig. 2Avi).

### Material characterization of OptoMaG

#### SEM and energy-dispersive x-ray spectroscopy

SEM imaging and energy-dispersive x-ray spectroscopy mapping of the OptoMaG were performed by a Zeiss Ultra 550 Gemini SEM. A segment of OptoMaG was taken and quickly cut with a sharp blade to obtain a cross section (fig. S1C). The cross section was placed on a sample stage inclined at 45° and coated with conductive adhesive. A 10-nm-thick gold layer was then sputtered onto the sample to reduce charging.

#### Triboindentation

Triboindentation experiments were carried out on OptoMaG samples (250-μm diameter, 10-mm length) with polished surfaces to determine the elastic modulus of the materials using a Hysitron TI-950 Triboindenter (Bruker Corporation, USA). During indentation, the indenter was advanced at a rate of 200 nm/s for nacre, prismatic calcite, and monolithic aragonite with a Berkovich tip (i.e., three-sided pyramidal diamond tip). The probe area function was calibrated for the Berkovich tip, particularly in the low-depth ranges, using a standard quartz sample before determining the mechanical properties accurately. Triboanalysis software was used to analyze complex data.

#### Optical characterization of OptoMaG

Two electrodes (the Ag-coated nylon wire and the copper wire) of a 30-cm-long guidewire were connected to an ac signal power source. The ac signal was generated using a Tektronix AFG 31000 series arbitrary function generator and amplified by a piezo amplifier (Model 2100HF, Trek Inc.). We can adjust the input voltage and frequency to modulate the luminescence intensity of the guidewire. The light power emitted from the guidewire was measured using a digital optical power meter and photodiode power sensor (Thorlabs PM100D, S120C). Compact charge-coupled device (CCD) spectrometers (Thorlabs CCS200/M) were used to measure the wavelength distribution and the emission color of the luminescence.

#### RF heating of magnetic tips

The OptoMaG with magnetic tips was loaded on the surface of A4 paper and irradiated with a RF heater (650 A, 350 kHz) at 1-, 5-, and 10-cm distances. Thermal images were obtained, and temperature information was recorded with a thermal infrared camera (ETS320, FLIR Systems) (*52*).

### Biomedical imaging

#### Micro-CT evaluation and analysis

After the macroscopic evaluation, the OptoMaG was scanned using a Bruker SkyScan 1276, with a high-resolution x-ray CCD camera (XIMEA MH110XC-KK-TP). Images were acquired at 10-μm resolution, at 85-kV source voltage, 200-μA source current, and a 1-mm aluminum filter. Scan duration was 3 hours 15 min. Images were reconstructed using Bruker NRecon and visualized using Bruker CTVox.

#### 2D x-ray imaging setup

An x-ray imaging device (XPERT 80, KUBTEC, Stratford, CT, USA) was used to visualize the OptoMaG shape changes in tissues. The x-ray accelerating voltage was set to 65 kV during the experiments. The x-ray dose was measured with the radiation meter (RM-400, Voltcraft GmbH) under the α + γ + β mode for the x-ray accelerating voltage of 65 kV and a current of 86 μA.

#### MRI of OptoMaG

MRI was performed on a 7 Tesla Bruker BioSpec, with an actively shielded gradient system (BGA20SHP) and 154-mm quadrature birdcage coil. A 2D gradient-echo imaging sequence (repetition time / echo time, 250/4 ms; 507 μm by 507 μm in-plane resolution; slice thickness, 0.5 mm, number of excitations, 2; total scan duration, 2 min 10 s) was used to image OptoMaG before and after insertion into the heart.

#### OA imaging of OptoMaG

*Comparison of imaging the OptoMaG and the commercial guidewire.* Tissue-mimicking imaging phantoms with a 2-cm-diameter were constructed from 1.5% (w/v) agar and 0.4% (w/v) Intralipid in distilled water. One cavity was created to facilitate the insertion of clear straws containing the OptoMaG and the commercial guidewire (ASAHI MEISTER 16, WAMS-165-1645). Then, the whole volume was inserted into the phantom cavity. The agar phantoms with inserts were imaged in the MSOT Imaging system (InVision 256-TF, iThera Medical, Munich, Germany). Specifically, the images were collected at 68 wavelengths (650 to 980 nm in 5-nm steps) at 25°C. Three image frames were measured per wavelength and averaged. This MSOT system is also capable of acquiring interleaved US images for the coregistration of imaging data. The US imaging signals of the OptoMaG were collected and analyzed using ViewMSOT software (version 4.1, iThera Medical GmbH, Munich, Germany). A 3D superimposition of OA and US images of the OptoMaG with magnetic tips was then constructed (Fig. 5, D and E).

*OA-guided navigation of OptoMaG through the different patterned soft tubes in deep tissues.* Different patterned soft tubes (SAI Infusion Technologies, 0.058 in. inner diameter (ID) × 0.077 in. outer diameter (OD), Sku # SIL-6-50) were embedded in 1.5% (w/v) agar and 0.4% (w/v) intralipid in distilled water. After the mixture solidified, the phantom was carefully placed above the MSOT imaging probe, taking care to avoid the formation of air bubbles. The setup was placed on an agar platform within the field of view of the MSOT imaging probe that was oriented upward to facilitate OptoMaG manipulation via a rotating magnet handheld by a robotic arm system. The robotic arm system for OptoMaG control includes a NEMA 17 stepper motor for magnet rotation and a 7-DOF robotic arm (Panda, Franka Emika GmbH).

*Pig heart for OptoMaG OA imaging ex vivo.* Ex vivo experiments were conducted under the permission of the regional ethical authority, Regierungspräsidium Stuttgart (registration no. DE08 111 1008 21). Pig organs and blood, sourced from a slaughterhouse, were refrigerated at 5°C and examined within 24 hours of receipt. The pig heart was used for validating the MSOT imaging performance. All organs were used as received, without any additional cleaning steps. A scalpel was used to section out tissue, incorporating the main and collateral vessels, allowing for the controlled egress of blood. A needle was inserted into a primary vessel branch for blood circulation through the phantom setup. The OptoMaG was inserted into the pig’s heart vasculature and manually manipulated to move forward, backward, and rotate. The setup was placed on an agar platform within the field of view of the MSOT imaging probe that was oriented upward to facilitate OptoMaG manipulation via a rotating magnet handheld by the robotic arm system. A specially fabricated blood retainer was used to prevent blood accumulation in the imaging field, thus minimizing the potential for significant imaging artifacts.

*Ex vivo OptoMaG OA imaging in the murine gastrointestinal system.* The BALB/c mice were freshly euthanized at the Institute of Comparative Molecular Endocrinology, Center for Biomedical Research, University of Ulm, according to §4 (*3*) TierSchG (animal welfare/protection law) (official permission number: MPIISS-UUL-o.189-31), and immediately used for further experiments. For ex vivo OA imaging experiments, the OptoMaG was inserted into the mouth and advanced through the gastrointestinal tract of the dead animal. The animal was scanned in the water tank of a 3D OA transducer (InVision 256-TF, iThera Medical, Munich, Germany), and the cross-sectional signals were collected at various sites along the gastrointestinal system.

### Cell culture experiments

#### Culture and differentiation of SH-SY5Y neuronal cells

SH-SY5Y cells were purchased from the Deutsche Sammlung von Mikroorganismen und Zellkulturen (American Type Culture Collection, CRL-2266, RRID: CVCL_0019). Maintenance cultures were grown in Dulbecco’s modified Eagle’s medium (DMEM)/F12 (Gibco, 11320033) with 10% fetal bovine serum (FBS) and 1% penicillin/streptomycin at 37°C with 5.0% CO_2_. The media was changed every 3 to 4 days. Before plating for experiments, wells were coated with laminin (5 μg/ml) in phosphate-buffered saline (PBS) with Ca^2+^/Mg^2+^ for 1 hour at 37°C. For toxicity analysis, cells were plated at a concentration of 20,000/cm^2^ onto cell culture–treated 96-well plates. Experimental cultures were differentiated in DMEM/F12 medium containing 1% FBS, 1% penicillin/streptomycin (Thermo Fisher Scientific, 15140122) and 10 μM retinoic acid (Sigma-Aldrich, R2625-50MG) for 4 days before all experiments.

#### RF-induced hyperthermia in in vitro conditions

The neuroblastoma cell line SH-SY5Y was seeded in a 96-well microplate (Thermo Fisher Scientific, 446612), and cells were cultured in three groups: control, OptoMaG’s body, and OptoMaG’s magnetic tips. The OptoMaG’s body group is that SH-SY5Y cells were cultured with the body of the OptoMaG without the magnetic tip. The OptoMaG’s magnetic tip group is that SH-SY5Y cells were cultured with complete OptoMaG with the magnetic tips, and the magnetic tips were put on the surface of the cells. These three groups were cultured for 72 hours in a humidified, 37°C, and 5% CO_2_ environment. These three groups were separately placed at a distance of 5 cm from the RF heater coil. The current in the RF heater coil was set at 650 A at a frequency of 350 kHz. Depending on the experiment, the RF heater coil was turned on for 0, 5, or 10 min. After RF heating, these three groups were collected and incubated in a humidified 37°C and 5% CO_2_ environment for 24 hours. The viabilities of SH-SY5Y cells were then measured using the LIVE/DEAD cell imaging kit assay (Thermo Fisher Scientific, R37601) according to the supplier’s instructions. A fluorescence microscope was used to identify live and dead cells. The luminescence values were measured in a 96-well plate using a TECAN Infinite M Plex microplate reader.

#### In vitro PDT

For functional tests of OptoMaG in vitro, SH-SY5Y cells were plated in cell culture–treated 24-well plates (Greiner, 662160) at a density of 50,000 cells/cm^2^. A total of 1 mM 5-ALA (Sigma-Aldrich, A7793) as photosensitizer was added to cells 24 hours later. The absorption of 5-ALA into the cells was monitored with a fluorescence microscope according to its spectrum. The culture medium was replaced with a fresh medium 4 hours later, and OptoMaG was applied to the cells for photostimulation. The stimulation was applied at an input power of 10 V and 50 kHz for 0, 1,2, 3, 4, 5, and 6 hours. After every time point during the stimulation, ROS is visualized with an dichlorodihydrofluorescein diacetate (H_2_DCFDA) assay (Thermo Fisher Scientific, D399) using fluorescence microscopy as described in a previous study (*53*).

#### Intracellular ROS measurement

At the end of the incubation, a final concentration of 25 μM of H_2_DCFDA (Abcam, ab113851) was added, and the cells were incubated at 37°C, with 5% CO_2_ in a humidified incubator for 30 min to allow loading of the dye. After 30 min, the cells were washed three times with PBS, and the conversion of nonfluorescent H_2_DCFDA into the highly fluorescent dichlorofluorescein was measured using fluorescence microscopy and the TECAN Infinite M Plex microplate reader. To define the background autofluorescence, nonstained cells were used. In addition, nontreated cells were used as controls.

#### WST-8 cell counting assay

Meanwhile, the WST-8 cell counting kit (Abcam, ab228554) was used to verify the photo effect according to the user manual. Briefly, ^1^/_10_ volume of WST-8 solution was added to the cell culture (100-μl WST-8 for a 24-well plate well with 1-ml medium). The plate with the reaction was kept in the incubator for 1 hour, and absorbance at 460 nm was measured with a TECAN Infinite M Plex microplate reader.

#### Accumulated intracellular PPIX quantification

The intracellular accumulation of PPIX in 5-ALA–treated cells was visualized using fluorescence microscopy. The fluorescence intensity of PPIX at 635 nm was measured following excitation at 400 nm, representing the accumulated PPIX. Images of PPIX fluorescence were captured using a Nikon spinning disk confocal microscope (Nikon Ti-E, Japan) at 0, 1, 2, 3, 4, and 5 hours after the addition of 5-ALA.

#### Statistical analyses

The sample size for each experiment is determined according to the a priori statistical power analysis. All experiments were repeated independently at least three times for replicability. Two experimental groups were tested with a *t* test and multiple experimental groups with one-way analysis of variance (ANOVA), and multiple experimental groups with several time points were statistically tested with two-way ANOVA. Quantitative data in figures were expressed as mean ± SD.

## Supplementary Materials

### The PDF file includes:

Supplementary Text Figs. S1 to S10 Tables S1 and S2 Legends for movies S1 to S6 References
