# Supplementary material for: Optoacoustically augmented magnetic guidewire for radiation-free minimally invasive therapies
Source: Sci Adv. 2026 Feb 4;12(6):eaea0201. doi: 10.1126/sciadv.aea0201 (PMC12871452; doi:10.1126/sciadv.aea0201)
Supplement: Supplementary file 1 — Supplementary Text Figs. S1 to S10 Tables S1 and S2 Legends for movies S1 to S6 References [file sciadv.aea0201_sm.pdf]

Science Advances
AAAS

Supplementary Materials for

Optoacoustically augmented magnetic guidewire for radiation-free minimally invasive therapies

Fan Wang *et al.*

Corresponding author: Erdost Yildiz, yildiz@is.mpg.de; Daniel Razansky, daniel.razansky@uzh.ch; Metin Sitti, msitti@ku.edu.tr

Sci. Adv. **12**, eaea0201 (2026)
DOI: 10.1126/sciadv.aea0201

The PDF file includes:

Supplementary Text
Figs. S1 to S10
Tables S1 and S2
Legends for movies S1 to S6
References

Other Supplementary Material for this manuscript includes the following:

Movies S1 to S6

Supplementary Materials

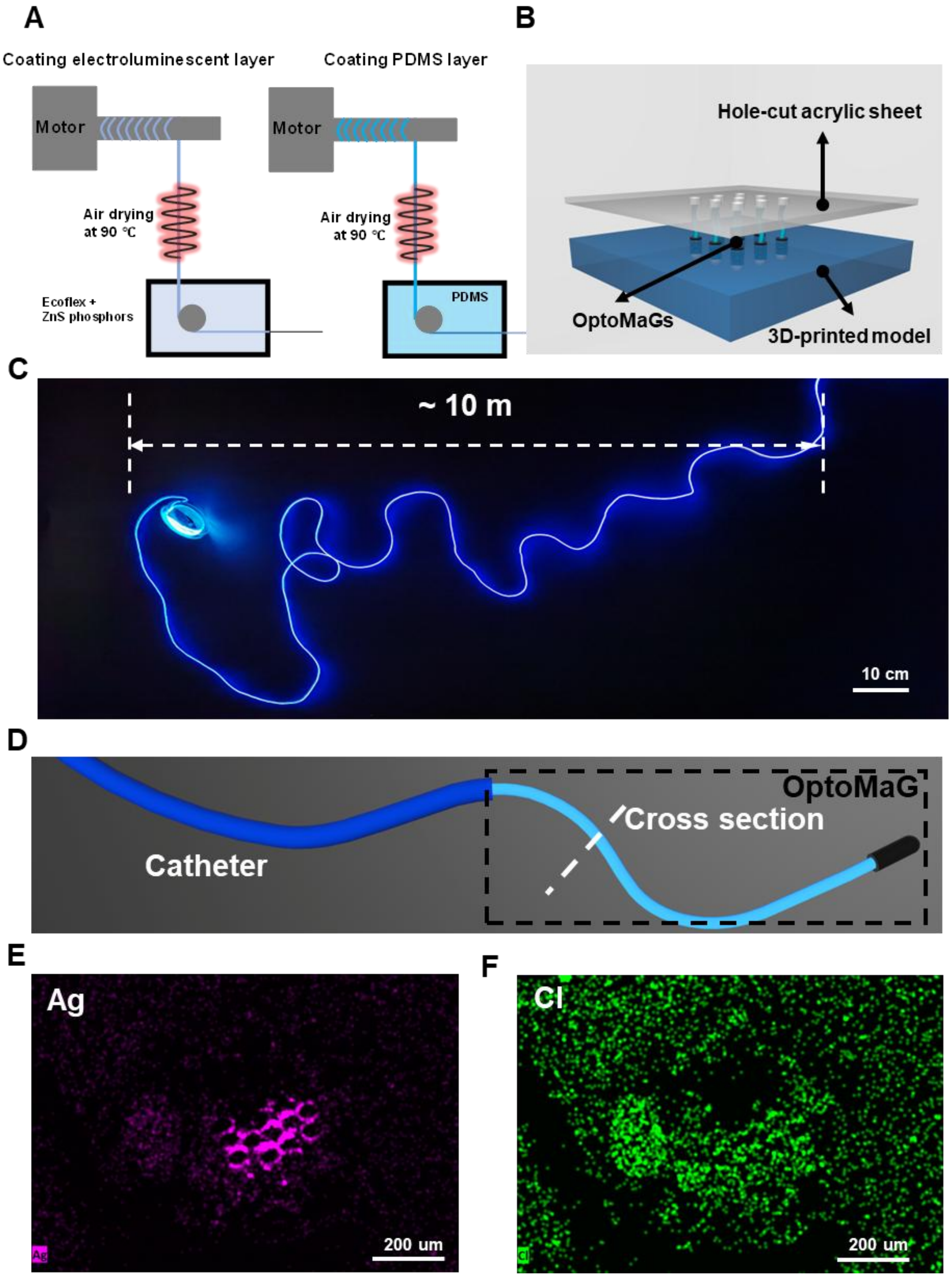

Fig. S1. OptoMaG's fabrication process and characterization: (A) Fabrication process of coating the electroluminescent and PDMS layer. (B) Schematic of the fabrication of the magnetic tip. (C) Photograph of a 10 m long OptoMaG with blue luminescence under AC power. (D)

Schematic of a single OptoMaG in a commercial catheter. (E) Ag distribution map on the cross-section of OptoMaG. (F) Cl distribution map on the cross-section of OptoMaG.

Supplementary Text 1: Luminescence mechanism of OptoMaG

In recent years, extensive research has been conducted on the AC electroluminescence (ACEL) mechanism of electroluminescent devices that utilize transition metal-doped zinc sulfides, such as ZnS: Cu. Among the various models proposed, Fisher's bipolar field-emission model is one of the most widely accepted theories *(54, 55).* While pure ZnS particles themselves are non-luminescent, they become effective donor-acceptor-type phosphors when doped with transition or rare earth metals. For instance, in the case of ZnS: Cu, Cl phosphor, the Cu activator functions as an acceptor, controlling the emission color, while Cl serves as an electron donor, facilitating charge injection into the phosphor layer *(55).*

During the preparation of ZnS powders for electroluminescent (EL) devices, lattice defects are often introduced, which promote the formation of conductive Cu2−xS needles within the ZnS crystal matrix. When an electric field is applied, the high field intensity becomes concentrated at the tips of these conductive needles. This intense electric field induces the tunneling of holes and electrons at both ends of the needle, leading to the generation of electron-hole pairs (*56*).

Electrons are subsequently trapped at the Cl donor sites, while holes are captured by the Cu acceptor sites. Upon field reversal, the recombination of electrons and holes results in light emission. In ACEL devices, the entire emissive layer contributes to light generation due to the uniform distribution of phosphor particles within the dielectric material. To generate light in electroluminescent devices, the applied voltage must be sufficiently high to accelerate electrons within the emissive layer. Typically, luminance values for AC ZnS powder EL devices range from 3 to 10 cd/m^2, and operating voltages generally exceed 100 V. Additionally, the input frequency significantly affects light brightness; however, the frequency must not surpass the electron lifetime, as charge carriers in the emissive layer may fail to recombine if the frequency is too high.

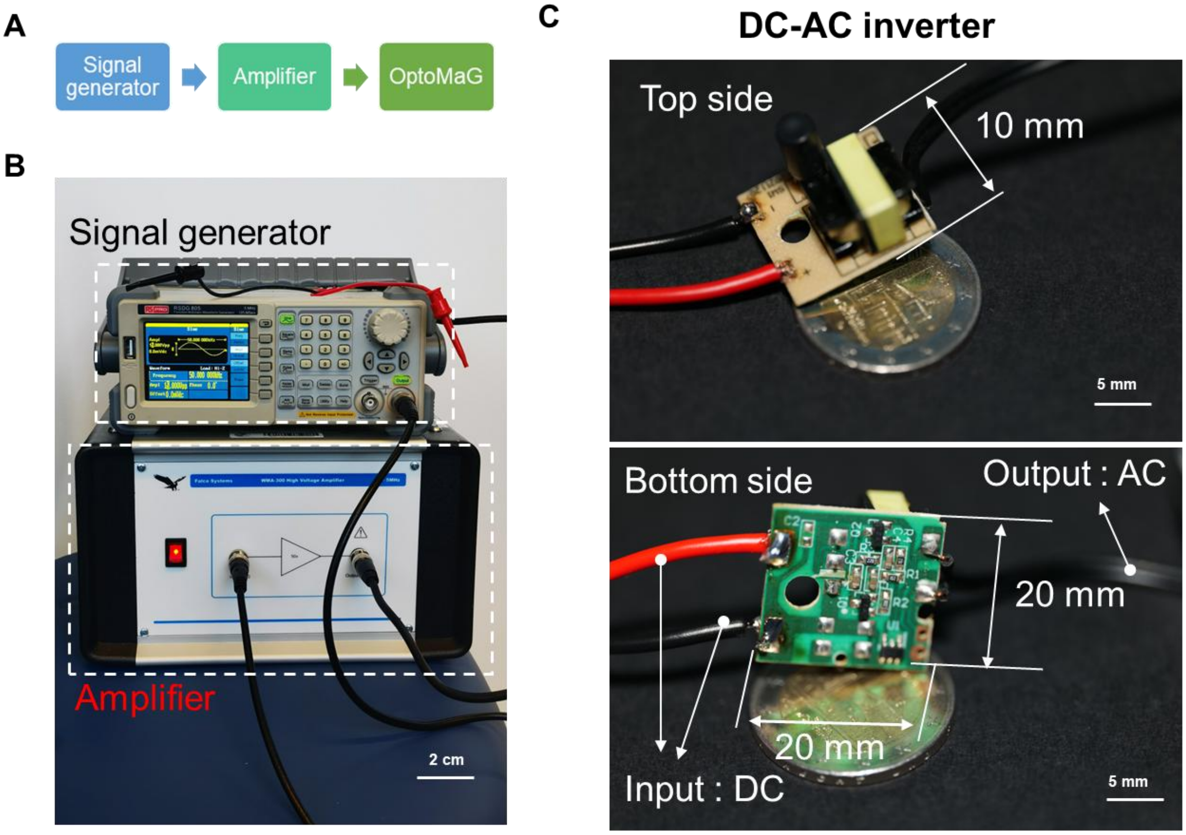

Fig. S2. Power electric system of OptoMaG: (A) Schematic of powering the OptoMaG. (B) Image of the experiment for characterization of luminescent OptoMaG, including a signal generator and an amplifier. (C) Portable and small-scale DC-AC circuit for powering the OptoMaG.

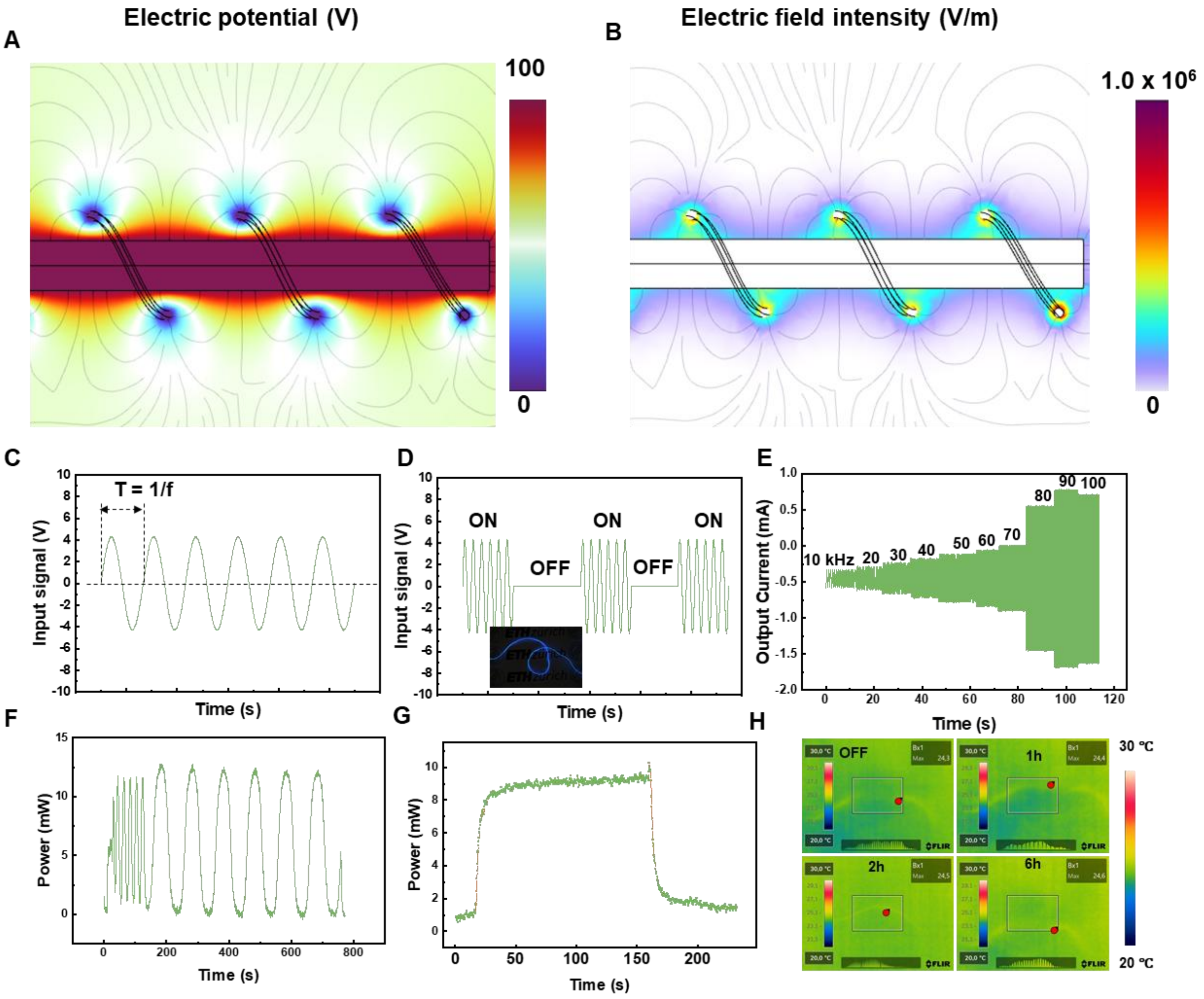

Fig. S3. Electric characterization of OptoMaG: (A, B) COMSOL simulation of electric potential and electric field intensity between two electrodes. (C) AC input signal. (D) Program the internal time between ON and OFF. (E) Output current of 30 cm OptoMaG at different input frequencies at 10 V. (F) Luminance power of OptoMaG at different frequencies. (G) Luminance power of OptoMaG in one cycle. (H) The temperature change of OptoMaG powered at different times (input voltage of 10V, frequency of 50 kHz)**.**

Supplementary Text 2: Mechanism of magnetic actuation of OptoMaG

To control OptoMaG within the human body, we employed magnetic fields due to their significant advantages in remote actuation, as shown in **Fig. 3A**. OptoMaG is internally embedded with magnetic particles in its head. According to electromagnetic principles, these particles experience

magnetic force $\boldsymbol{F_m}$ and torque $\boldsymbol{T_m}$ when subjected to an external magnetic field, which are respectively given by:

$$\boldsymbol{F_m} = \int_V (\boldsymbol{m} \cdot \boldsymbol{\nabla})\boldsymbol{B} dV \quad \text{(S1)}$$

$$\boldsymbol{T_m} = \int_V (\boldsymbol{m} \times \boldsymbol{\nabla})\boldsymbol{B} dV \quad \text{(S2)}$$

where $\boldsymbol{F_m}$ is the force acting on the magnetic particles embedded in OptoMaG, $\boldsymbol{m}$ means the internal magnetization, $\boldsymbol{B}$ represents the magnetic flux density, V is the volume of the magnetic material, and $\boldsymbol{T_m}$ means the torque acting on the magnetic particles embedded in OptoMaG. Free space, $\boldsymbol{B}$, can be further expressed as follows (*55*):

$$\boldsymbol{B} = \boldsymbol{\mu_0 H} \quad \text{(S3)}$$

where $\boldsymbol{\mu}_0$ is the magnetic permeability of free space, which has a constant value of $4\pi\times10^{-7}$ N·A^{-2}, and $\boldsymbol{H}$ is the magnetic field strength (A m^{-1}).

Assuming the magnetic particles are uniformly embedded within the material, we simplify the aforementioned equations by utilizing the magnetic moment $\boldsymbol{M}$ of the magnetic material for calculations. The magnetic moment $\boldsymbol{M}$ can be measured using a vibrating sample magnetometer (VSM). Consequently, the force and torque acting on the magnetic material in a magnetic field can be calculated as follows:

$$\boldsymbol{F_m} = (\boldsymbol{M} \cdot \boldsymbol{\nabla})\boldsymbol{B} \quad \text{(S4)}$$

$$\boldsymbol{T_m} = \boldsymbol{M} \times \boldsymbol{B} \quad \text{(S5)}$$

Based on the aforementioned principles, we placed OptoMaG in a magnetic field. The head of OptoMaG contains uniformly distributed magnetic particles that experience both force and torque. In a uniform magnetic field, it is primarily subjected to magnetic torque, as shown in **Fig. 4B**. Under the influence of the magnetic field, the head of OptoMaG deforms to the left, causing the body of OptoMaG to bend as well. This bending deformation enables us to control the shape change of OptoMaG inside the body by applying an external magnetic field. Consequently, it becomes possible to manipulate OptoMaG within bodily lumens, such as navigating and selecting specific vascular branches. To verify the bending behavior of OptoMaG under a magnetic field, we conducted simulations using ABAQUS based on the aforementioned equations. In the simulation, to simplify the calculations, we assumed that OptoMaG was initially suspended

vertically with gravity acting downward. A uniform magnetic field directed to the left was then applied, and the resulting deformation was calculated. Based on this setup, we obtained the simulation results shown in **Fig. 4C**. Additionally, we performed experiments to test the deformation of OptoMaG under the same conditions as the simulation, utilizing the VSM to generate various constant magnetic fields. The experimental results are presented in **Fig. 4D**.

To enable a more intuitive comparison, we employed two parameters to describe the deformation: the minimum radius of curvature, r_{min} , and the tip deflection angle, θ (**Fig. 4B**). We used circles of different radii to fit the contour of OptoMaG, obtaining various radius values, r_i , and the minimum radius among these was selected as a key parameter. The tip deflection angle is defined as the angle between the axis of the OptoMaG head and the vertical direction. Using MATLAB, we calculated these values for OptoMaG under different magnetic fields for both the simulation and the experiments, as shown in **Fig. 4E** and **Fig. S4A**. We found excellent consistency between the simulation and experimental results, thereby demonstrating the feasibility of precisely controlling OptoMaG in practical applications using the proposed method.

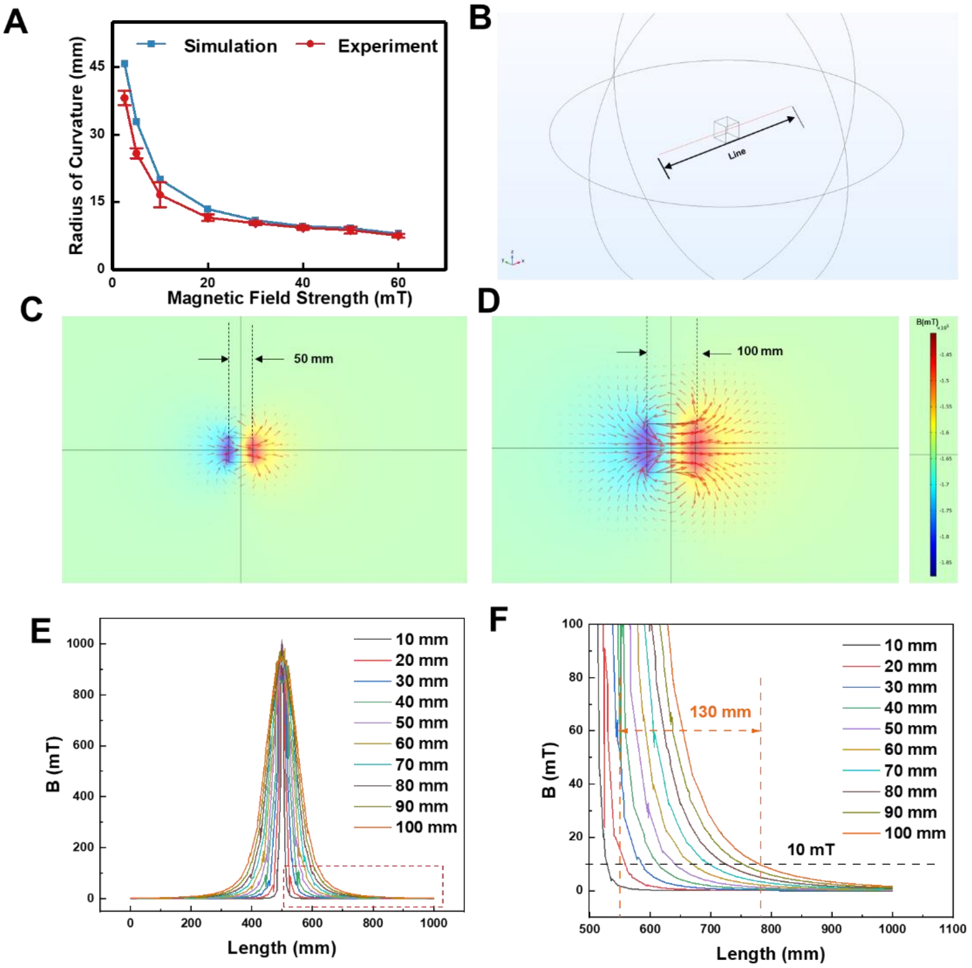

Fig. S4. Mechanism of magnetic actuation of OptoMaG: (A) Comparison of the radius of curvature under varying magnetic fields between simulation and experimental results. (B) COMSOL model of a single magnetic cube. (C, D) Spatial distribution of the magnetic field surrounding magnetic cubes with dimensions of 50 mm × 50 mm × 50 mm and 100 mm × 100 mm × 100 mm, respectively. (E, F) Magnetic field distribution along a line for different sizes of magnetic cubes.

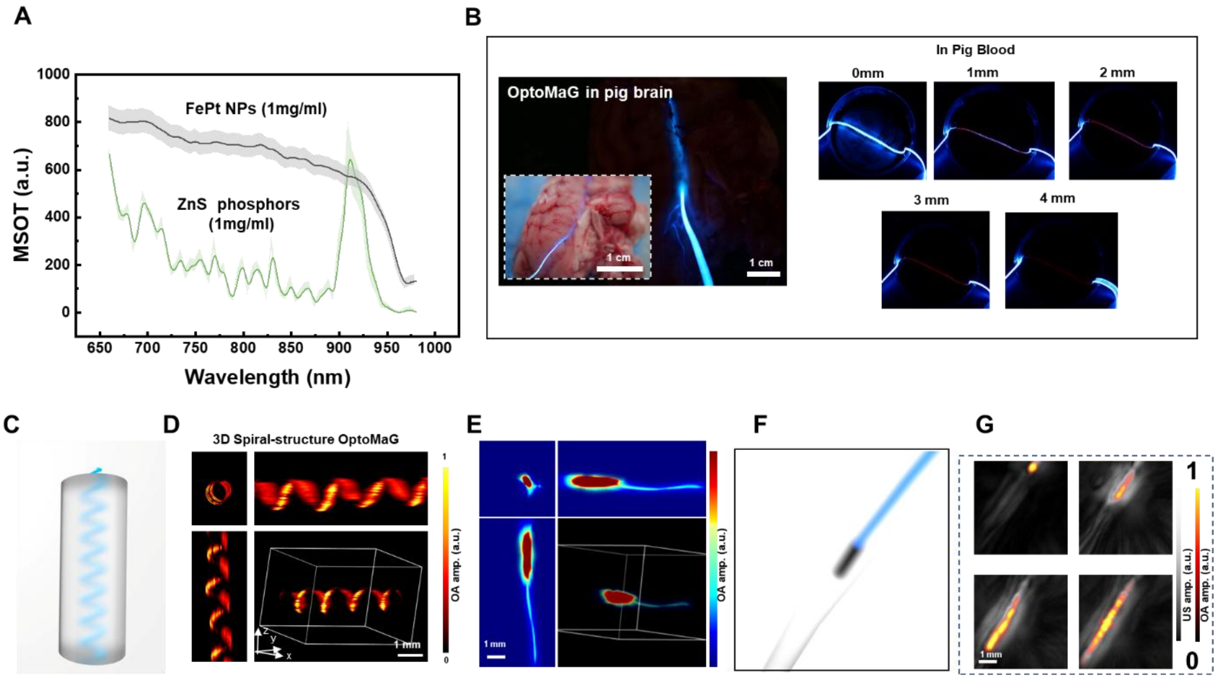

Fig. S5. OA imaging properties of the OptoMaG: (A) The spiral-shaped OptoMaG is embedded within the Agarose hydrogel. (B) OA images of a 3D Spiral-structure OptoMaG without magnetic tips in X, Y, and Z projections, respectively, and a 3D reconstructed image. (C) OA images of OptoMaG with magnetic tips in the Agarose hydrogel. (D) OA spectra of 1 mg/ml FePt NPs solution and 1 mg/ml ZnS phosphors solution. (E) Images of luminescent OptoMaG in different depths of fresh pig blood. (F) Schematic of OptoMaG going through a soft tube. (G) OptoMaG is going through a soft tube with real-time OA imaging guidance.

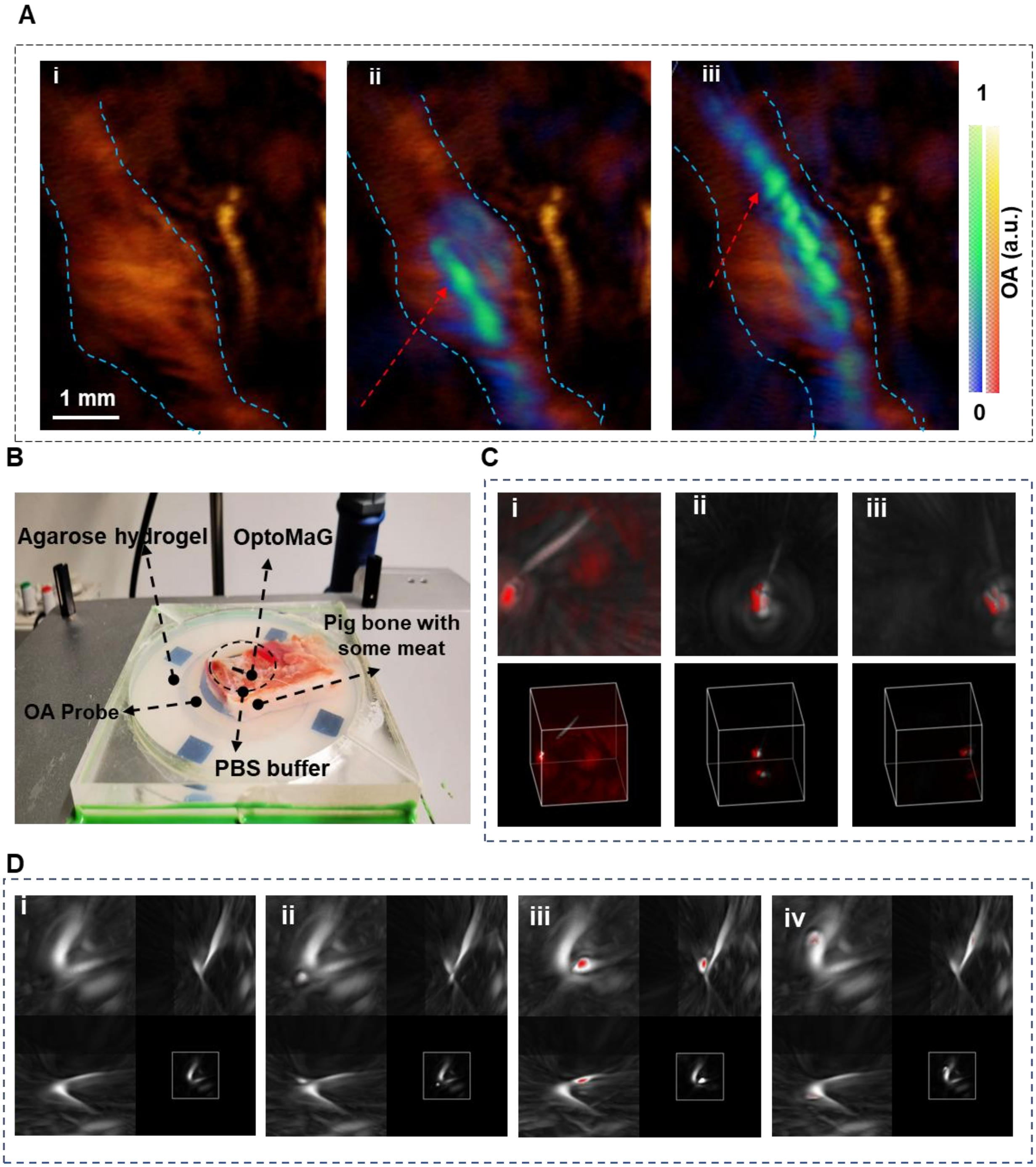

Fig. S6. Optoacoustic imaging setup for OptoMaG: (A) OA images acquired before(i), during(ii), and after(iii) the insertion of OptoMaG into the blood vessel at different laser wavelengths of 800nm (red), 920nm (green). The green image is a differential image, i. e. Image minus background. Background subtraction in the MSOT imaging process revealed the OptoMaG signal with enhanced contrast, enabling clear delineation of vascular structures against the blood

background. (B) Experiment setup for testing the bending behaviors of OptoMaG responding to the magnetic field. (C) Bending behaviors of OptoMaG responding to the magnetic field. (D) OptoMaG intelligently moves through the Y-shaped soft tube by controlling it.

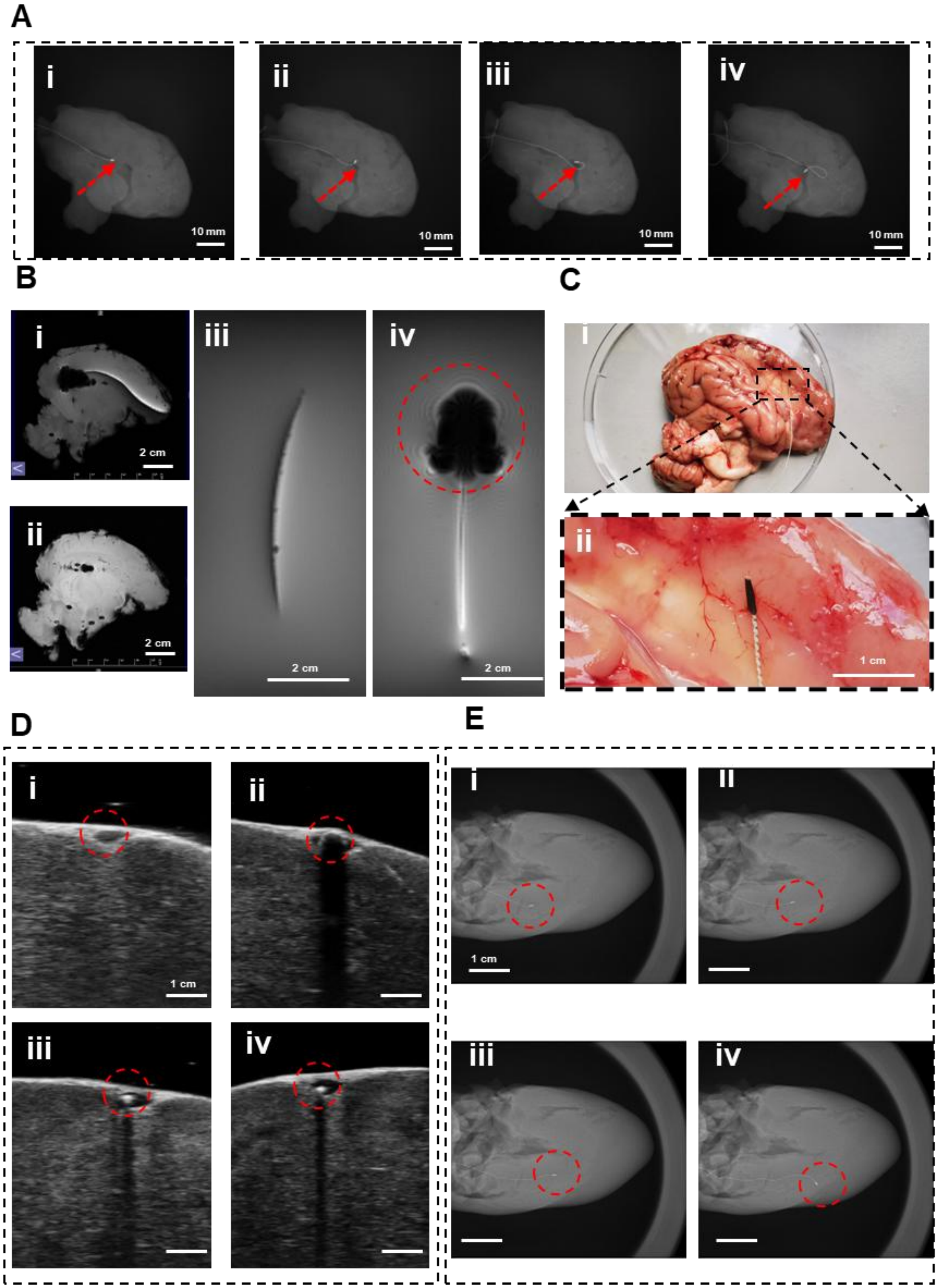

Fig. S7. OptoMaG movement in various organs in different medical imaging technologies. (A) 3D OA and US imaging of OptoMaG in a tube. (B) X-ray images of OptoMaG moving in the

porcine brain. (C) MRI images of OptoMaG in the porcine brain. (D) Real-time image of OptoMaG in the porcine brain. (E) US imaging OproMaG moving in the porcine heart. (F) X-ray images of OptoMaG moving in the pig heart.

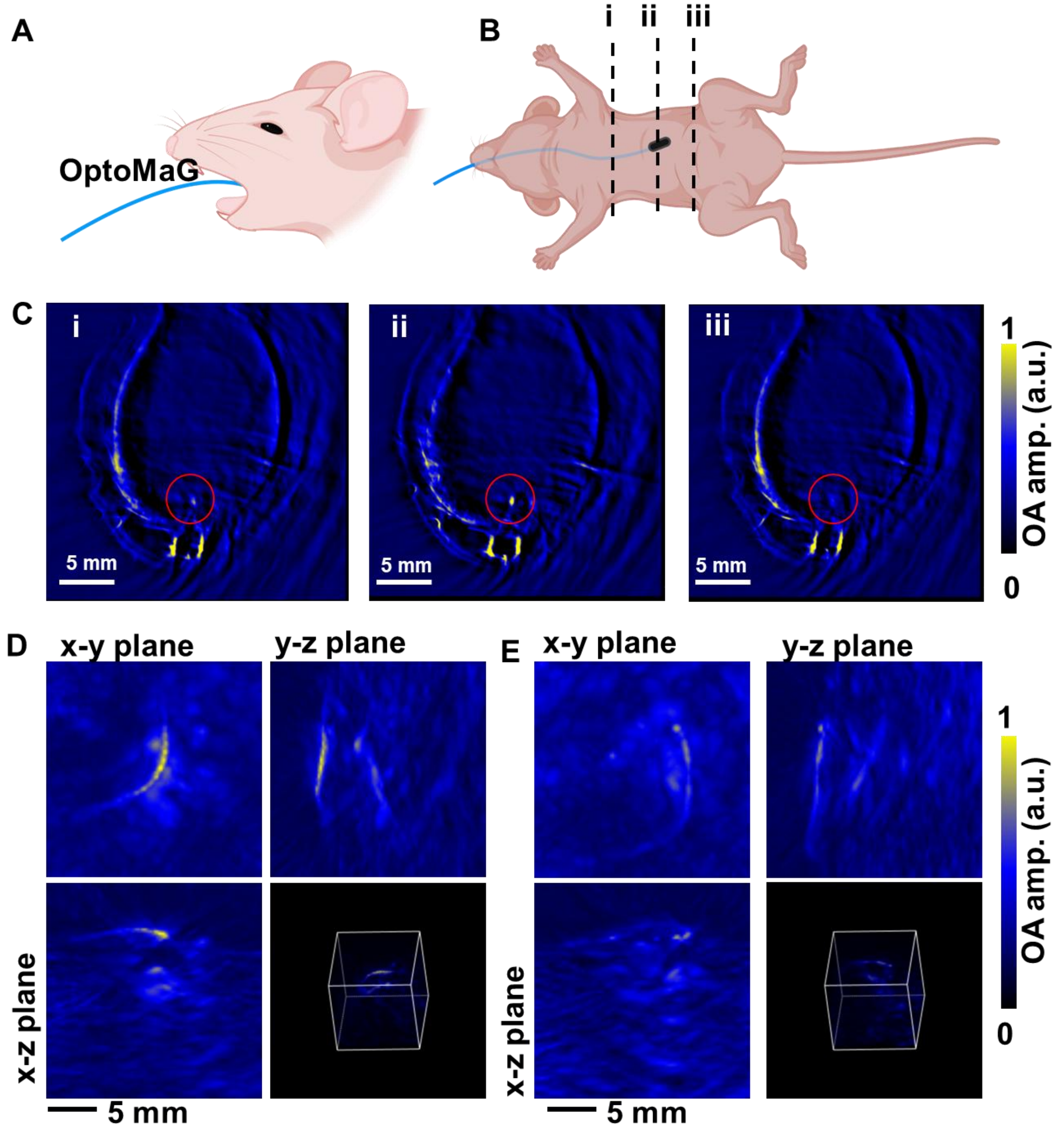

Fig. S8. Ex vivo OptoMaG OA imaging in the murine gastrointestinal system: (A and B) An OptoMaG was put into the mouse's body, and OA images were taken at different anatomical locations. (C) OA imaging the OptoMaG at the cross-section of the mouse body (i, ii, iii). (D and E) 3D images of OptoMaG moving through the mouse body using the handle 3D probe.

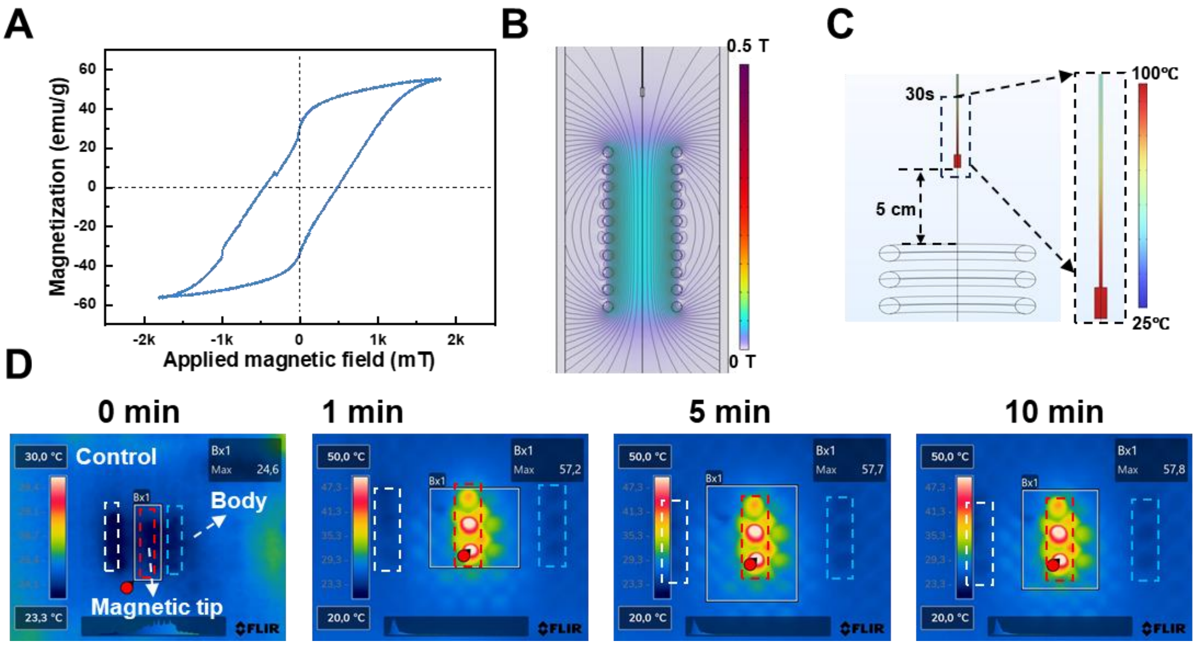

Fig. S9. Magnetic and radio-frequency heating characterization: (A) Hysteresis loops of FePt particles. The curves are normalized by dividing the maximum magnetic moment value obtained under 1.8 T. (B and C) COMSOL simulation of RF induction heating magnetic tip of OptoMaG. (D) RF hyperthermia experiment for the SH-SY5Y cell in a 96-well plate in three groups: control, magnetic tip, and body.

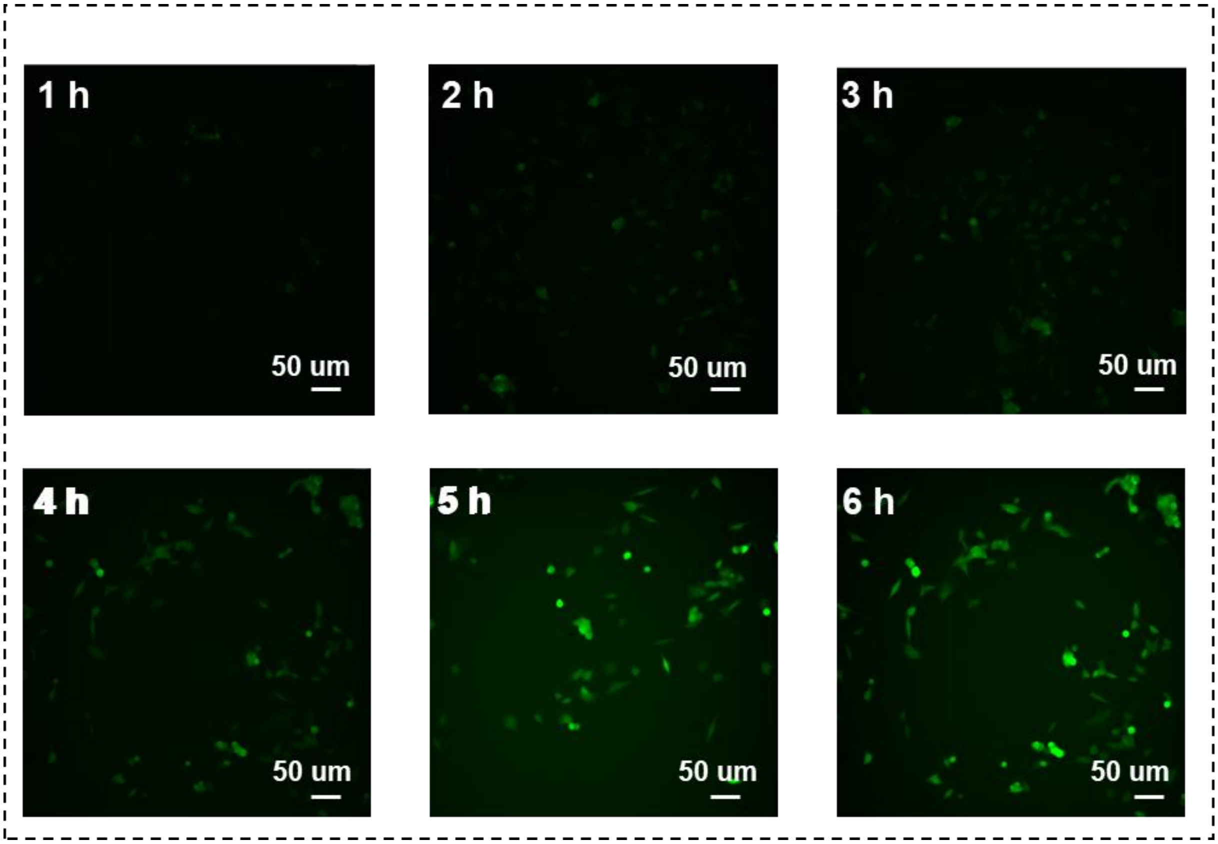

Fig. S10. ROS generation in different light simulation times.

Table S1. Summary of the key advantages and disadvantages of various imaging modalities in observing and detecting medical guidewires (*3*).

**Imaging Modality**	**Type of energy measurement**	**Imaging Depth**	**Temporal Resolution**	**Spatial Resolution**	**Ionizing radiation**	**Contrasting agent**	**Advantages**	**Disadvantages**
MRI	Radio waves	> 10 cm	< 0.1 Hz	10 – 100 μm	No	Yes	1. High contrast and clarity for soft tissues<br>2. No ionizing radiation<br>3. Multiplanar imaging capability	1. Material restrictions (metallic artifacts)<br>2. Need contrast agent<br>3. Long imaging time<br>4. Complex and expensive equipment
US	Double-directional ultrasound waves	≈10 cm	10 – 10000 Hz	100 - 500 μm	No	No	1. Real-time imaging<br>2. No radiation<br>3. Portable and easy to operate	1. Operator-dependent image quality<br>2. Limited penetration depth<br>3. Lower soft tissue contrast
X-ray	High-energy gamma rays	> 10 cm	< 0.1 Hz	50 – 500 μm	Yes	Yes	1. Quick imaging<br>2. High spatial resolution	1. Ionizing radiation exposure<br>2. Low soft tissue contrast

							3. Widely available	3. Primarily 2D imaging
OA Imaging	Single-direction ultrasound waves	7 cm	10 – 100 Hz for a 3D image		No	No,	1. No ionizing radiation<br>2. High contrast for specific tissues (e.g., blood vessels) without contrasting agents.<br>3. Multifunctional imaging (combined with ultrasound)<br>4. 3D Real-time imaging	1. Limited clinical validation<br>2. Limited imaging depth<br>3. Long Processing Times for Spatial Reconstruction

Table S2. Comparison of OptoMaG and conventional guidewires across different imaging modalities in terms of spatial/temporal resolution, imaging depth, field of view, and contrast *(57–60)*.

Imaging Modality	Metric	OptoMaG	Conventional Guidewires (ASAHI MEISTER 16, WAMS-165-1645)
**Optoacoustic (OA)**	**Spatial resolution**	~120 µm in deep tissues	Not visible/negligible OA contrast
	**Temporal resolution**	Up to 10 Hz for 3D imaging	— (Not detectable)
	**Imaging depth**	Up to 10 mm in porcine brain; up to ~12 cm in phantoms (depends on the structure of the detector)	—
	**Field of view**	Moderate (limited by MSOT probe geometry)	— (Not applicable)
	**Contrast**	Strong OA signal due to ZnS:Cu and FePt composite	Poor; typically no detectable signal
**Ultrasound (US)**	**Spatial resolution**	100–500 µm	100–500 µm
	**Temporal resolution**	Real-time (10–10,000 Hz)	Real-time (10–10,000 Hz)
	**Imaging depth**	~10 cm	~10 cm
	**Field of view**	Cross-sectional (probe-dependent)	Cross-sectional (probe-dependent)
	**Contrast**	Moderate (via selective absorption enhancement)	Moderate; metallic backscatter may cause artifacts
**X-ray / CT**	**Spatial resolution**	~500 µm (FePt tip visible at high exposure)	Excellent (~100–200 µm) with contrast agents
	**Temporal resolution**	Static/low frame rate	Static/low frame rate
	**Imaging depth**	Whole-body	Whole-body
	**Field of view**	Wide	Wide
	**Contrast**	Weak without a metal or contrast agent	Good with contrast agents; requires ionizing radiation
**MRI**	**Spatial resolution**	10–100 µm (FePt tip causes susceptibility artifacts)	Moderate; signal distortion from metallic tips (non-metallic guidewire)
	**Temporal resolution**	Slow (typically seconds per frame)	Slow

	**Imaging depth**	Deep (whole-brain achievable)	Deep
	**Field of view**	Whole-body	Whole-body
	**Contrast**	Limited due to magnetic interference	Poor; artifact-prone in the presence of metal

Other Supplementary Materials for this manuscript include the following:

Movie S1. ON-OFF response of the guidewire at various frequencies.
We used a signal generator to program the output voltage, enabling the OptoMaG to be powered at various frequencies.

Movie S2. Deformation of the OptoMaG under varying magnetic fields.
We used a vibrating-sample magnetometer (VSM) to generate different uniform magnetic fields, under which the vertically downward-oriented OptoMaG underwent varying degrees of deformation due to the different magnitudes of magnetic force.

Movie S3. Movement control of the guidewire in Y-shaped tubing.
We controlled the OptoMaG with external magnetic fields to selectively navigate through different branches of the Y-shaped tube.

Movie S4. Movement control of the guidewire in a 3D vascular phantom of the human brain.
With the assistance of a commercial catheter, the OptoMaG was delivered into the cerebral vasculature and subsequently guided by external magnetic fields to the target site, where it provided localized blue-light illumination to deep brain regions.

Movie S5. Movement control for the guidewire through a C-shaped tubing.
We employed optoacoustic imaging to real-time monitor the passage of OptoMaG through the C-shaped tubing.

Movie S6. RF-induced heating function of the guidewire.
The OptoMaG was positioned 5 cm above the RF heater, and upon activation of the RF source, the temperature of its magnetic head gradually increased.

REFERENCES

1. D. B. Buck, J. A. van Herwaarden, M. L. Schermerhorn, F. L. Moll, Endovascular treatment of abdominal aortic aneurysms. *Nat. Rev. Cardiol.* **11**, 112–123 (2014).

2. B. Hasan, M. Farah, T. Nayfeh, M. Amin, K. Malandris, R. Abd-Rabu, S. Shah, R. Rajjoub, M. O. Seisa, S. Saadi, L. Hassett, L. J. Prokop, A. F. AbuRahma, M. H. Murad, A systematic review supporting the Society for Vascular Surgery Guidelines on the management of carotid artery disease. *J. Vasc. Surg.* **75**, 99S–108S.e42 (2022).

3. U. Bozuyuk, P. Wrede, E. Yildiz, M. Sitti, Roadmap for clinical translation of mobile microrobotics. *Adv. Mater.* **36**, e2311462 (2024).

4. T. Wang, Y. Wu, E. Yildiz, S. Kanyas, M. Sitti, Clinical translation of wireless soft robotic medical devices. *Nat. Rev. Bioeng.* **2**, 470–485 (2024).

5. R. Nunna, F. Tariq, F. Jummah, N. Bains, A. I. Qureshi, F. Siddiq, Advances in the endovascular management of cerebrovascular disease. *Mo. Med.* **121**, 127–135 (2024).

6. Y. Yan, T. Wang, R. Zhang, Y. Liu, W. Hu, M. Sitti, Magnetically assisted soft milli-tools for occluded lumen morphology *detection*. *Sci.Adv.* **9**, eadi3979 (2023).

7. T. Wang, H. Ugurlu, Y. Yan, M. Li, M. Li, A.-M. Wild, E. Yildiz, M. Schneider, D. Sheehan, W. Hu, M. Sitti, Adaptive wireless millirobotic locomotion into distal vasculature. *Nat. Commun.* **13**, 4465 (2022).

8. S. Laksono, R. M. R. J. Pasciolly, H. Munirwan, A. P. Pratama, A. S. Prawara, Choosing the appropriate catheter and wire in peripheral intervention. *AsiaIntervention* **8**, 162–170 (2022).

9. A. Roguin, J. Goldstein, O. Bar, J. A. Goldstein, Brain and neck tumors among physicians performing interventional procedures. *Am. J. Cardiol.* **111**, 1368–1372 (2013).

10. M. Kamran, A. N. Wallace, A. Adewumi, Interventional management of head and neck tumors. *Semin. Intervent. Radiol.* **37**, 157–165 (2020).

11..n lü, K. Deloose, V. B. M. E. Mees, Guidewire selection and techniques in peripheral arterial interventions. *Eur. J. Vasc. Endovasc. Surg.* **67**, 1027–1030 (2024).

12. B. Buchberger, K. Scholl, L. Krabbe, L. Spiller, B. Lux, Radiation exposure by medical x-ray applications. *Ger. Med. Sci.* **20**, Doc06 (2022).

13. M. Andreucci, R. Solomon, A. Tasanarong, Side effects of radiographic contrast media: Pathogenesis, risk factors, and prevention. *Biomed. Res. Int.* **2014**, 741018 (2014).

14. T. Salam, L. Wilson, S. Bohannan, M. Morin, Safety and effectiveness of a novel fluoroless transseptal puncture technique for lead-free catheter ablation: A case series. *J. Innov. Cardiac. Rhythm Manag.* **11**, 4079–4085 (2020).

15. X. L. Deán-Ben, S. Gottschalk, B. M. Larney, S. Shoham, D. Razansky, Advanced optoacoustic methods for multiscale imaging of in vivo dynamics. *Chem. Soc. Rev.* **46**, 2158–2198 (2017).

16. A. C. Stiel, V. Ntziachristos, Controlling the sound of light: Photoswitching optoacoustic imaging. *Nat. Methods* **21**, 1996–2007 (2024).

17. M. Eleni Karakatsani, H. Estrada, Z. Chen, S. Shoham, X. L. Deán-Ben, D. Razansky, Shedding light on ultrasound in action: Optical and optoacoustic monitoring of ultrasound brain interventions. *Adv. Drug Deliv. Rev.* **205**, 115177 (2024).

18. D. Razansky, A. Buehler, V. Ntziachristos, Volumetric real-time multispectral optoacoustic tomography of biomarkers. *Nat. Protoc.* **6**, 1121–1129 (2011).

19. E. Yildiz, U. Bozuyuk, E. Yildiz, F. Wang, M. Han, A. C. Karacakol, D. Sheehan, Y. Yu, M. Sitti, Magnetically controllable and degradable milliscale swimmers as intraocular drug implants. *Adv. Sci.* **12**, e07569 (2025).

20. N. Driscoll, M.-J. Antonini, T. M. Cannon, P. Maretich, G. Olaitan, V. D. P. Van, K. Nagao, A. Sahasrabudhe, E. V. Paniagua, E. J. Frey, Y. J. Kim, S. Hunt, M. Hummel, S. Mupparaju, A. Jasanoff, B. J. Venton, P. Anikeeva, Multifunctional neural probes enable bidirectional

electrical, optical, and chemical recording and stimulation in vivo. *Adv. Mater.* **37**, 2408154 (2025).

21. Y. Kim, G. A. Parada, S. Liu, X. Zhao, Ferromagnetic soft continuum robots. *Sci. Robot.* **4**, eaax7329 (2019).

22. T. S. Mang, Lasers and light sources for PDT: past, present and future. *Photodiagnosis Photodyn. Ther.* **1**, 43–48 (2004).

23. Z. Wang, Y. Liu, Z. Zhou, P. Chen, H. Peng, Towards integrated textile display systems. *Nat. Rev. Electr. Eng.* **1**, 466–477 (2024).

24. H. Peng, "Fiber light-emitting devices" in *Fiber Electronics*, H. Peng, Ed. (Springer, 2020), pp. 253–289.

25. K. Mahmoudi, K. Garvey, A. Bouras, G. Cramer, H. Stepp, J. Jesu Raj, D. Bozec, T. Busch, C. Hadjipanayis, 5-Aminolevulinic acid photodynamic therapy for the treatment of high-grade gliomas. *J. Neurooncol* **141**, 595–607 (2019).

26. J. Schanda, *Colorimetry: Understanding the CIE System* (John Wiley & Sons, 2007).

27. J. F. Algorri, M. Ochoa, P. Roldán-Varona, L. Rodríguez-Cobo, J. M. López-Higuera, Light technology for efficient and effective photodynamic therapy: A critical review. *Cancer* **13**, 3484 (2021).

28. K. Zhang, X. Shi, H. Jiang, K. Zeng, Z. Zhou, P. Zhai, L. Zhang, H. Peng, Design and fabrication of wearable electronic textiles using twisted fiber-based threads. *Nat. Protoc.* **19**, 1557–1589 (2024).

29. J. H. Park, S. K. Park, T. H. Kim, J. J. Shin, H. S. Shin, Y. S. Hwang, Anterior communicating artery aneurysm related to visual symptoms. *J Korean Neurosurg Soc.* **46**, 232–238 (2009).

30. R. H. Soon, Z. Yin, M. A. Dogan, N. O. Dogan, M. E. Tiryaki, A. C. Karacakol, A. Aydin, P. Esmaeili-Dokht, M. Sitti, Pangolin-inspired untethered magnetic robot for on-demand biomedical heating applications. *Nat. Commun.* **14**, 3320 (2023).

31. M. Szwed, A. Marczak, Application of nanoparticles for magnetic hyperthermia for cancer treatment—The current state of knowledge. *Cancer* **16**, 1156 (2024).

32. D. Kessel, Photodynamic therapy: Critical PDT theory. *Photochem. Photobiol.* **99**, 199–203 (2023).

33. Z. Zhou, J. Song, L. Nie, X. Chen, Reactive oxygen species generating systems meeting challenges of photodynamic cancer therapy. *Chem. Soc. Rev.* **45**, 6597–6626 (2016).

34. M. Roshanfar, M. Salimi, A. H. Kaboodrangi, S.-J. Jang, A. J. Sinusas, S.-C. Wong, B. Mosadegh, Advanced robotics for the next-generation of cardiac interventions. *Micromachines* **16**, 363 (2025).

35. M. E. Tiryaki, Y. G. Elmacıoğlu, M. Sitti, Magnetic guidewire steering at ultrahigh magnetic fields. *Sci. Adv.* **9**, eadg6438 (2023).

36. S. Mallidi, G. P. Luke, S. Emelianov, Photoacoustic imaging in cancer detection, diagnosis, and treatment guidance. *Trends Biotechnol.* **29**, 213–221 (2011).

37. J. F. Algorri, J. M. López-Higuera, L. Rodríguez-Cobo, A. Cobo, Advanced light source technologies for photodynamic therapy of skin cancer lesions. *Pharmaceutics* **15**, 2075 (2023).

38. "IEEE standard for safety levels with respect to human exposure to electric, magnetic, and electromagnetic fields, 0 Hz to 300 GHz - Redline," in *IEEE Std C95.1-2019 (Revision of IEEE Std C95.1-2005/ Incorporates IEEE Std C95.1-2019/Cor 1-2019) - Redline* (IEEE, 2019), pp. 1–679.

39. J. D. Wells, S. Thomsen, P. Whitaker, E. D. Jansen, C. C. Kao, P. E. Konrad, A. Mahadevan-Jansen, Optically mediated nerve stimulation: Identification of injury thresholds. *Lasers Surg. Med.* **39**, 513–526 (2007).

40. R. Gandhi, C. Tsoumpas, Preclinical imaging biomarkers for postischaemic neurovascular remodelling. *Contrast Media Mol. Imaging* **2019**, 3128529 (2019).

41. M. Graham, F. Assis, D. Allman, A. Wiacek, E. Gonzalez, M. Gubbi, J. Dong, H. Hou, S. Beck, J. Chrispin, M. A. L. Bell, In vivo demonstration of photoacoustic image guidance and robotic visual servoing for cardiac catheter-based interventions. *IEEE Trans. Med. Imaging* **39**, 1015–1029 (2020).

42. X. Yang, C. Chai, Y.-H. Chen, M. Sawan, Skull impact on photoacoustic imaging of multi-layered brain tissues with embedded blood vessel under different optical source types: Modeling and simulation. *Bioengineering* **12**, 40 (2025).

43. J. Robin, C. Demené, B. Heiles, V. Blanvillain, L. Puke, F. Perren, M. Tanter, *In vivo* adaptive focusing for clinical contrast-enhanced transcranial ultrasound imaging in human. *Phys. Med. Biol.* **68**, 025019 (2023).

44. S. Zhou, X. Gao, G. Park, X. Yang, B. Qi, M. Lin, H. Huang, Y. Bian, H. Hu, X. Chen, R. S. Wu, B. Liu, W. Yue, C. Lu, R. Wang, P. Bheemreddy, S. Qin, A. Lam, K. A. Wear, M. Andre, E. B. Kistler, D. W. Newell, S. Xu, Transcranial volumetric imaging using a conformal ultrasound patch. *Nature* **629**, 810–818 (2024).

45. H. Estrada, A. Özbek, J. Robin, S. Shoham, D. Razansky, Spherical array system for high-precision transcranial ultrasound stimulation and optoacoustic imaging in rodents. *IEEE Trans. Ultrason. Ferroelectr. Freq. Control* **68**, 107–115 (2021).

46. Y. Wang, Y. Chen, Y. Gao, M. Zhang, Q. Cheng, "Transcranial ultrasound focusing with flexible array transducer" in *2024 IEEE Ultrasonics, Ferroelectrics, and Frequency Control Joint Symposium (UFFC-JS)* (IEEE, 2024), pp. 1–4.

47. X. L. Dean-Ben, A. Buehler, V. Ntziachristos, D. Razansky, Accurate model-based reconstruction algorithm for three-dimensional optoacoustic tomography. *IEEE Trans. Med. Imaging* **31**, 1922–1928 (2012).

48. A. Paul, S. Mallidi, Enhancing signal-to-noise ratio in real-time LED-based photoacoustic imaging: A comparative study of CNN-based deep learning architectures. *Photoacoustics* **41**, 100674 (2025).

49. X. Luís Dean-Ben, D. Razansky, Localization optoacoustic tomography. *Light Sci. Appl.* **7**, 18004–18004 (2018).

50. Y. Jiang, H. J. Lee, L. Lan, H. Tseng, C. Yang, H.-Y. Man, X. Han, J.-X. Cheng, Optoacoustic brain stimulation at submillimeter spatial precision. *Nat. Commun.* **11**, 881 (2020).

51. I. Dimaridis, P. Sridharan, V. Ntziachristos, A. Karlas, L. Hadjileontiadis, Image quality improvement techniques and assessment adequacy in clinical optoacoustic imaging: A systematic review. *Biosensors* **12**, 901 (2022).

52. S. Ersoy, E. Yıldız, Z. Ren, M. Zhang, H. Zhang, S. Karaz, M. Han, A. Shiva, M. Yunusa, C. Kaya, Fabrication of gold nanoflower-coated photosensitive meta-structures using PμSL 3D printing for hyperthermia applications. *ACS Appl. Polym. Mater.* **6**, 10807–10823 (2024).

53. O. Karatum, E. Yildiz, H. N. Kaleli, A. Sahin, B. Ulgut, S. Nizamoglu, RuO_2 supercapacitor enables flexible, safe, and efficient optoelectronic neural interface. *Adv. Funct. Mater.* **32**, 2109365 (2022).

54. A. G. Fischer, Electroluminescent lines in ZnS powder particles: I . Embedding media and basic observations. *J. Electrochem. Soc.* **109**, 1043 (1962).

55. Y. Y. Chen, J. G. Duh, B. S. Chiou, C. G. Peng, Luminescent mechanisms of ZnS:Cu:Cl and ZnS:Cu:Al phosphors. *Thin Solid Films* **392**, 50–55 (2001).

56. X. Wang, H. Zhang, R. Yu, L. Dong, D. Peng, A. Zhang, Y. Zhang, H. Liu, C. Pan, Z. L. Wang, Dynamic pressure mapping of personalized handwriting by a flexible sensor matrix based on the mechanoluminescence process. *Adv. Mater.* **27**, 2324–2331 (2015).

57. J. Hwang, J. Kim, H. Choi, A review of magnetic actuation systems and magnetically actuated guidewire- and catheter-based microrobots for vascular interventions. *Intell. Serv. Robot.* **13**, 1–14 (2020).

58. P. Wrede, E. Remlova, Y. Chen, X. L. Deán-Ben, M. Sitti, D. Razansky, Synergistic integration of materials in medical microrobots for advanced imaging and actuation. *Nat. Rev. Mater.* **10**, 888–906 (2025).

59. X. Ju, C. Chen, C. M. Oral, S. Sevim, R. Golestanian, M. Sun, N. Bouzari, X. Lin, M. Urso, J. S. Nam, Y. Cho, X. Peng, F. C. Landers, S. Yang, A. Adibi, N. Taz, R. Wittkowski, D. Ahmed, W. Wang, V. Magdanz, M. Medina-Sánchez, M. Guix, N. Bari, B. Behkam, R. Kapral, Y. Huang, J. Tang, B. Wang, K. Morozov, A. Leshansky, S. A. Abbasi, H. Choi, S. Ghosh, B. B. Fernandes, G. Battaglia, P. Fischer, A. Ghosh, B. J. Sánchez, A. Escarpa, Q. Martinet, J. Palacci, E. Lauga, J. Moran, M. A. Ramos-Docampo, B. Städler, R. S. H. Restrepo, G. Yossifon, J. D. Nicholas, J. Ignés-Mullol, J. Puigmartí-Luis, Y. Liu, L. D. Zarzar, C. W. I. Shields, L. Li, S. Li, X. Ma, D. H. Gracias, O. Velev, S. Sánchez, M. J. Esplandiu, J. Simmchen, A. Lobosco, S. Misra, Z. Wu, J. Li, A. Kuhn, A. Nourhani, T. Maric, Z. Xiong, A. Aghakhani, Y. Mei, Y. Tu, F. Peng, E. Diller, M. S. Sakar, A. Sen, J. Law, Y. Sun, A. Pena-Francesch, K. Villa, H. Li, D. E. Fan, K. Liang, T. J. Huang, X.-Z. Chen, S. Tang, X. Zhang, J. Cui, H. Wang, W. Gao, V. K. Bandari, O. G. Schmidt, X. Wu, J. Guan, M. Sitti, B. J. Nelson, S. Pané, L. Zhang, H. Shahsavan, Q. He, I.-D. Kim, J. Wang, M. Pumera, Technology roadmap of micro/nanorobots. *ACS Nano* **19**, 24174–24334 (2025).

60. D. Li, Y. Zhang, C. Liu, J. Chen, D. Sun, L. Wang, Review of photoacoustic imaging for microrobots tracking in vivo. *Chin. Opt. Lett.* **19**, 111701 (2021).
